# Electrode Colonization by the Feammox Bacterium *Acidimicrobiaceae* sp. Strain A6

**DOI:** 10.1128/AEM.02029-18

**Published:** 2018-11-30

**Authors:** Melany Ruiz-Urigüen, Weitao Shuai, Peter R. Jaffé

**Affiliations:** a Department of Civil and Environmental Engineering, Princeton University, Princeton, New Jersey, USA; Wageningen University

**Keywords:** *Acidimicrobiaceae* sp. A6, *Actinobacteria*, Feammox, ammonium oxidation, anaerobic, electrode-reducing bacteria, iron reduction, lithoautotrophic, wetland soils

## Abstract

*Acidimicrobiaceae* sp. strain A6 (A6), from the *Actinobacteria* phylum, was recently identified as a microorganism that can carry out anaerobic ammonium (NH_4_^+^) oxidation coupled to iron reduction, a process also known as Feammox. Being an iron-reducing bacterium, A6 was studied as a potential electrode-reducing bacterium that may transfer electrons extracellularly onto electrodes while gaining energy from NH_4_^+^ oxidation. *Actinobacteria* species have been overlooked as electrogenic bacteria, and the importance of lithoautotrophic iron reducers as electrode-reducing bacteria at anodes has not been addressed. By installing electrodes in the soil of a forested riparian wetland where A6 thrives, in soil columns in the laboratory, and in A6-bioaugmented constructed wetland (CW) mesocosms and by operating microbial electrolysis cells (MECs) with pure A6 culture, the characteristics and performances of this organism as an electrode-reducing bacterium candidate were investigated. In this study, we show that *Acidimicrobiaceae* sp. strain A6, a lithoautotrophic bacterium, is capable of colonizing electrodes under controlled conditions. In addition, A6 appears to be an electrode-reducing bacterium, since current production was boosted shortly after the CWs were seeded with enrichment A6 culture and current production was detected in MECs operated with pure A6, with the anode as the sole electron acceptor and NH_4_^+^ as the sole electron donor.

**IMPORTANCE** Most studies on electrogenic microorganisms have focused on the most abundant heterotrophs, while other microorganisms also commonly present in electrode microbial communities, such as *Actinobacteria* strains, have been overlooked. The novel *Acidimicrobiaceae* sp. strain A6 (*Actinobacteria*) is an iron-reducing bacterium that can colonize the surface of anodes in sediments and is linked to electrical current production, making it an electrode-reducing bacterium. Furthermore, A6 can carry out anaerobic ammonium oxidation coupled to iron reduction. Therefore, findings from this study open the possibility of using electrodes instead of iron as electron acceptors, as a means to promote A6 to treat NH_4_^+^-containing wastewater more efficiently. Altogether, this study expands our knowledge of electrogenic bacteria and opens the possibility of developing Feammox-based technologies coupled to bioelectric systems for the treatment of NH_4_^+^ and other contaminants in anoxic systems.

## Abstract (precis)

Most studies on electrogenic microorganisms have focused on the most abundant heterotrophs, while other microorganisms also commonly present in electrode microbial communities, such as *Actinobacteria* strains, have been overlooked. The novel *Acidimicrobiaceae* sp. strain A6 (*Actinobacteria*) is an iron-reducing bacterium that can colonize the surface of anodes in sediments and is linked to electrical current production, making it an electrode-reducing bacterium. Furthermore, A6 can carry out anaerobic ammonium oxidation coupled to iron reduction. Therefore, findings from this study open the possibility of using electrodes instead of iron as electron acceptors, as a means to promote A6 to treat NH_4_^+^-containing wastewater more efficiently. Altogether, this study expands our knowledge of electrogenic bacteria and opens the possibility of developing Feammox-based technologies coupled to bioelectric systems for the treatment of NH_4_^+^ and other contaminants in anoxic systems.

## INTRODUCTION

Electrode-reducing bacteria (ERB) are part of a group of electrogenic microorganisms that have the ability to extract energy from different types of electron donors, such as organic matter, and transfer those electrons to various terminal electron acceptors, including electrodes operating as anodes, in which case a low-density electrical current is produced (1). Known electrogenic microorganisms include yeast (*Pichia anomala*) and various bacteria (2). Studies on community composition analysis of ERB show ample taxonomic diversity, mostly dominated by three phyla, *Firmicutes*, *Acidobacteria*, and *Proteobacteria*; the latter contains some of the most commonly present and extensively studied ERB, namely, *Geobacter* spp. and *Shewanella* spp. (1–4). Most of these organisms are heterotrophs that thrive in anaerobic environments and obtain their energy by oxidizing organic matter (1). ERB commonly are iron-reducing bacteria (FeRB) (1), and many depend on or benefit from electron shuttles to facilitate the transfer of electrons from the microorganism to a solid electron acceptor, such as the iron oxides (5).

*Acidimicrobiaceae* sp. strain A6 (referred to as A6) is an autotrophic anaerobic microorganism that obtains its energy by oxidizing ammonium (NH_4_^+^) to nitrite (NO_2_^−^) and transferring the electrons to oxidized iron [Fe(III)], which acts as the final electron acceptor under environmental conditions (6, 7), in a process known as Feammox (8–10). Similar to other metal-reducing bacteria, *Acidimicrobiaceae* sp. strain A6, a type of actinobacteria, has the ability to use other sources of electron acceptors (11). The *Actinobacteria* phylum is commonly present in microbial community composition analyses of biomass associated with electrodes (12–17), but its role on the electrodes is rarely analyzed, most likely because it is not among the most abundant phyla. To the best of our knowledge, to date there is only one report of electrogenic actinobacteria, i.e., heterotrophic bacteria of the genus *Dietzia* that were isolated from an intertidal zone of the Río de la Plata (18). A6 is an iron reducer (7) that can use anthraquinone-2,6-disulfonate (AQDS), a humic acid analogue, as an electron shuttle (10, 19). These characteristics of A6 raised the possibility of it also being a type of ERB. Although A6 was isolated and grown under strict anaerobic conditions (10), A6 enrichment cultures were shown to exhibit Feammox activity under anoxic conditions (dissolved oxygen [DO] levels of <0.8 mg/liter) (20); the exact DO levels that A6 cells in those experiments might have been exposed to in microlocations within the slurry are not known and might have been much lower.

Lithoautotrophs are microorganisms that use inorganic compounds as their energy sources and CO_2_ as their carbon source. These types of microorganisms are usually studied as part of the communities that develop at the cathode, because they can be electrotrophs, i.e., they can take up electrons directly from the cathode as their energy source, and they thrive on the CO_2_ formed by the oxidation of organic matter by ERB (21, 22). The microbial communities that develop at the cathode are as highly diverse as the communities that develop at the anode, and many of the microbial groups found at the anode as ERB have also been found at the cathode (17, 23), some of them proven electrotrophs, including *Geobacter* species (22, 24). Among the phyla present at both the anode and the cathode, *Actinobacteria* can usually be found.

Microbial fuel cells (MFCs) and microbial electrolysis cells (MECs) are two reactor configurations that utilize electrogenic microorganisms for renewable energy production, bioelectricity generation, and pollutant degradation. In particular, MFCs coupled to constructed wetlands (CWs) have been used as devices to explore the possibility of treating wastewater and producing electricity simultaneously (25, 26). With the incorporation of MFCs into planted CWs, MFC operations can be promoted by the oxygen excreted by plant roots (27), resulting in stratified redox conditions that develop in wetland soils (28).

Given that the Feammox process has been found in multiple submerged sediments (7, 8, 29–31) and that A6 was isolated from wetland sediments (20), studying A6 in the field and in CWs is advantageous for understanding and characterizing this bacterium. The objectives of this study were (i) to investigate whether *Acidimicrobiaceae* sp. strain A6, like other electrogenic bacteria, could colonize electrodes placed in sediments under natural or controlled conditions and thus could be enriched with respect to the surrounding sediments and (ii) to analyze the ability of A6 to transfer electrons to an electrode, by determining current increments in electrode-containing CWs and MECs in the presence of *Acidimicrobiaceae* sp A6. These findings expand the knowledge of the diversity of ERB by including a member of the previously overlooked phylum *Actinobacteria*, and they allow for the possibility of practical applications of *Acidimicrobiaceae* sp. strain A6 for the treatment of NH_4_^+^ contamination in anoxic systems, using electrodes as stable, long-term, terminal electron acceptors.

## RESULTS

### *Acidimicrobiaceae* sp. strain A6 quantification. (i) A6 quantification in the field study.

Bacterial counting by quantitative PCR (qPCR) confirmed our initial hypothesis that the counts of A6 could be enhanced on the electrode surface, because the bacteria may have the ability to use electrodes as electron acceptors in the same manner that other FeRB do. The average count of A6 among the bacteria attached to the electrodes from the first group incubated for 52 days was orders of magnitude greater than the average count of the bacterium in the soil (4.13 × 10^6^ copies of DNA/m^2^ on the electrode and 7.46 × 10^3^ copies of DNA/m^2^ in the soil). The second group was maintained for 32 days, and the results showed that A6 levels quantified from bacteria attached to the electrode were significantly higher than those from soils (Welch *t* test, *P* < 0.05), unlike the controls, for which no significant difference was found.

**FIG 1:**
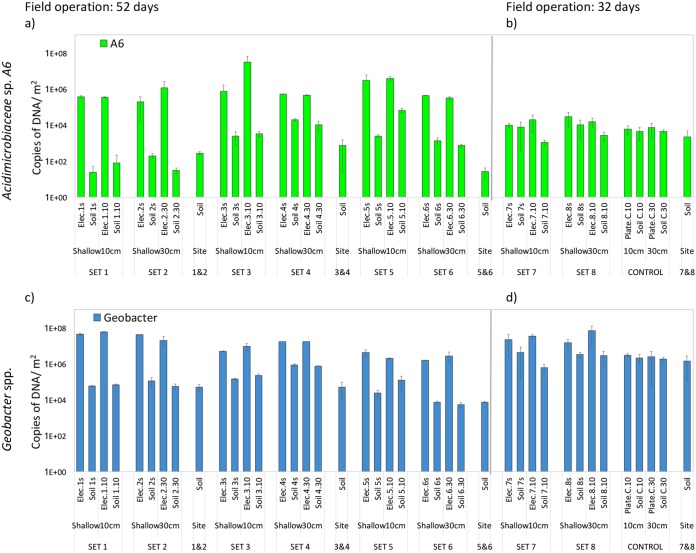
Quantification of *Acidimicrobiaceae* sp. strain A6 and *Geobacter* spp. from bacteria attached to connected electrodes and control unconnected graphite plates and from soil samples. (a and b) *Acidimicrobiaceae* sp. strain A6 quantification after 52 days in field operation (a) and after 32 days in field operation (b). (c and d) *Geobacter* spp. quantification after 52 days in field operation (c) and after 32 days in field operation (d). Bars represent the mean and error bars the standard deviation (SD) (*n* = 3).

In order to confirm that the electrodes were also being colonized by other electrogenic bacteria, we choose to quantify the genus *Geobacter*, which has become a model organism for the study of electrogenic organisms. *Geobacter* spp. also had significant greater populations among the bacteria attached to the surface of the electrodes, compared with the surrounding soil (Welch *t* test, *P* < 0.001) (Fig. 1). On average, across all sites, the quantification of A6 and *Geobacter* spp. resulted in higher bacterial counts on the electrodes than in the surrounding soil but not on the unconnected graphite plates (Table 1). These results indicated that the numbers of A6 were clearly enhanced by the electrodes.

**TABLE 1:** Average numbers of copies of DNA quantified for *Acidimicrobiaceae* sp. strain A6 and *Geobacter* spp. from bacteria attached to the electrodes and in soil samples from field and laboratory sets, as well as current measured for each set at the time of retrieval

Set	No. of copies of DNA/m^2^						*I* (µA)
	*Acidimicrobiaceae* sp. strain A6			*Geobacter* spp.			
	Electrode	Soil	Site	Electrode	Soil	Site	
Field							
1	3.65 × 10^5^	5.15 × 10^1^	2.73 × 10^2^	5.12 × 10^7^	6.32 × 10^4^	5.09 × 10^4^	40.45
2	6.64 × 10^5^	1.13 × 10^2^		3.06 × 10^7^	8.16 × 10^4^		0.40
3	1.59 × 10^7^	2.85 × 10^3^	7.51 × 10^2^	7.13 × 10^6^	1.84 × 10^5^	5.04 × 10^4^	2.00
4	4.85 × 10^5^	1.51 × 10^4^		1.68 × 10^7^	8.04 × 10^5^		0.75
5	3.40 × 10^6^	3.37 × 10^4^	2.60 × 10^1^	3.09 × 10^6^	7.15 × 10^4^	7.32 × 10^3^	0.66
6	3.72 × 10^5^	1.04 × 10^3^		2.12 × 10^6^	6.22 × 10^3^		−0.50
7^*a*^	1.46 × 10^4^	4.33 × 10^3^	2.16 × 10^3^	2.77 × 10^7^	2.42 × 10^6^	1.42 × 10^6^	35.2 ± 26
8^*a*^	2.27 × 10^4^	6.45 × 10^3^		4.07 × 10^7^	3.01 × 10^6^		62.9 ± 48
C^*a*^^,^^*b*^	6.49 × 10^3^	4.40 × 10^3^		2.72 × 10^6^	1.94 × 10^6^		0.00
Laboratory							
9	1.36 × 10^3^	8.17 × 10^1^		9.66 × 10^6^	6.98 × 10^5^		113.0
C	1.15 × 10^2^	8.17 × 10^1^		4.65 × 10^6^	8.05 × 10^5^		0.00

a Average from triplicate samples.

b C, control, unconnected, graphite plates.

**FIG 2:**
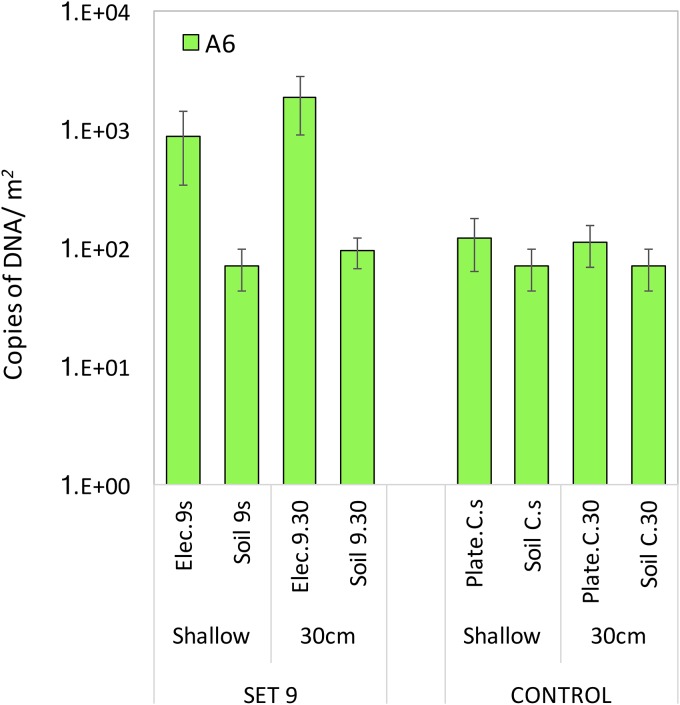
*Acidimicrobiaceae* sp. strain A6 quantification from bacteria attached to the connected electrodes (Elec.) and control unconnected graphite plates during 52 days of incubation in soil columns in the laboratory. Bars represent the mean and error bars the SD (*n* = 4).

However, we could not always see a trend in biomass being greater on the deeper electrodes than on the shallower electrodes in the field, as hypothesized. We had initially assumed that the surroundings of the shallow electrode would be more oxidized and thus it would act as the cathode, with the deep electrode being more reduced and thus acting as the anode. Because the first group of electrodes was placed in the field for 52 days and the second group for 32 days, without any interference, during which multiple days of rain were recorded, with as much as 21.8 mm in July 2016 and 81.5 mm in May 2018 (see Fig. S1 in the supplemental material), the redox state of the soils might have shifted, thus inverting the polarity of the electrodes due to fluctuations in the water table. Such conditions might have favored the colonization of ERB on both electrodes (deep and shallow) at different times. Current measurements were taken between the deep and shallow electrodes at the time of their retrieval (Table 1). All sets showed positive current except set 6, which had a current of −0.50 µA, thus indicating that electrode polarity might have shifted while the electrodes were in the field. Therefore, the soil cores taken from the field to the laboratory were used in soil columns to test a set of connected electrodes (set 9), as well as a set of unconnected graphite plates as controls. Under laboratory conditions, the soil columns were kept saturated with water for the duration of the incubations, to avoid changes in the redox conditions due to water level fluctuations. The incubations carried out in the laboratory resulted in higher A6 counts on the deep electrode than on the shallow one, and both electrodes had greater biomass than the surrounding soil (Welch *t* test, *P* < 0.01) (Fig. 2). The control graphite plates showed no statistically significant difference between the amount of A6 attached to the unconnected electrodes and the amount in the surrounding soil.

### (ii) A6 quantification in the CW mesocosm study.

**FIG 3:**
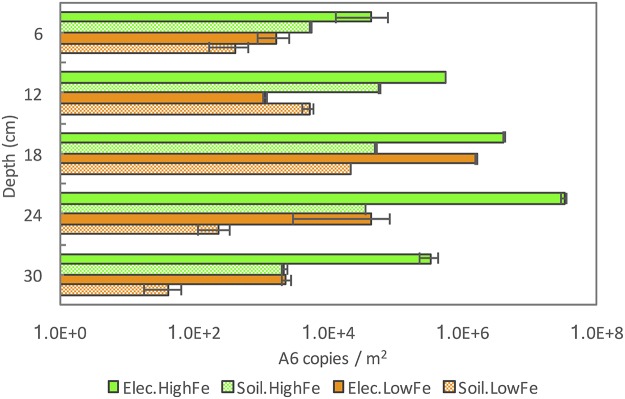
*Acidimicrobiaceae* sp. strain A6 populations in the CW sediments (Soil) and at the electrodes (Elec.) in CW mesocosms. Error bars are the upper and lower values of qPCR measurements.

The populations of A6 are shown in Fig. 3 for all samples from both CW mesocosms. The A6 population was greater in the high-Fe mesocosm than in the low-Fe mesocosm, either on the CW sediments or among bacteria attached to the electrodes (*P* < 0.05). Furthermore, the amount of A6 attached to the electrode was always 2 to 3 orders of magnitude greater than that in the surrounding sediments for the deeper electrodes (depths of >15 cm) in both mesocosms.

The oxidation-reduction potential (ORP) profile and the current between electrode pairs were monitored in the CWs to clarify which electrodes were working as the cathodes and which as the anodes. Electrode 1 (at a depth of 6 cm) was designated the cathode at the beginning but, as the redox potential profile in the CW developed, the direction of the current between electrode 1 and electrode 2 (at a depth of 12 cm) reversed around day 35. Therefore, on day 59, electrode 2 was designated the cathode and the wires were reconnected to form electrode pairs between electrode 2 and other electrodes. Figure S2 shows that the ORP at the location of electrode 2 was highest before the injection of A6 enrichment culture. Because the electrodes below 15 cm (electrodes 3 to 5) had lower ORPs throughout the entire experiment, those electrodes were always operated as the anodes. A tendency for A6 colonization on the anodes was observed (for A6 counts on the electrode versus in the soil at a depth of >15 cm, *P* < 0.05), whereas the two shallower electrodes, which each operated as the cathode for a certain period, did not always show larger numbers of A6, compared to the surrounding soil, and no statistically significant difference in the count numbers for those samples was found (for A6 counts on the electrode versus in the soil at a depth of <15 cm, *P* > 0.05). These results confirmed the higher affinity of A6 for the electrode (anode) that remained in the more reduced soil throughout the experiment than for the electrode (cathode) in the more oxidized soil. Therefore, these well-controlled and monitored CW mesocosms could provide insights that were not clearly resolved in the field studies.

### (iii) Current production, NH_4_^+^ removal, and A6 quantification in MECs.

**FIG 4:**
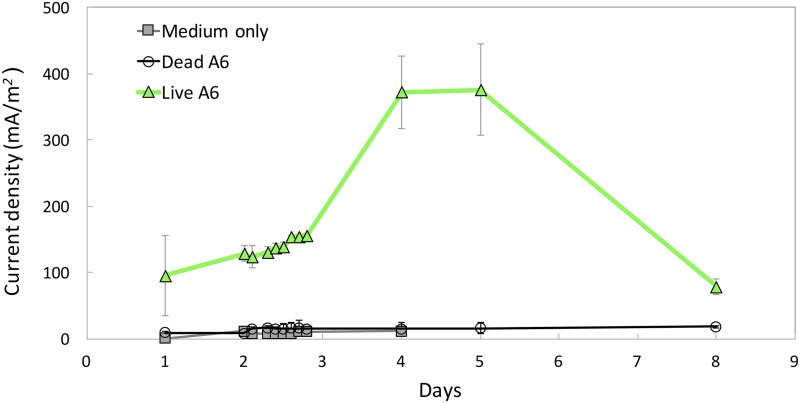
*I_d_* measured in MECs operated with a pure A6 culture, with dead A6, and with no A6 inoculated. Markers show the mean and error bars the SD.

MECs inoculated with pure A6 culture showed clear current production, with a maximum current density (*I_d_*) of 376.1 mA/m^2^ (Fig. 4). MECs containing dead A6 culture or Feammox medium only had no to very low *I_d_*, with an average of 8.4 ± 5.8 mA/m^2^. No NH_4_^+^ concentration change was detected in MECs containing medium only or dead bacteria, while an average of 1.14 ± 0.2 mM NH_4_^+^ removal was measured for the MECs operated with live A6. Finally, A6 cell counts increased from 5 × 10^4^ copies of DNA/ml to 9.8 × 10^5^ copies of DNA/ml after the whole operation period. Through scanning electron microscopy, bacterial cells attached to the surface of the anode were detected (Fig. S3), with an estimated total of 2.8 × 10^8^ cells/m^2^; in the control MECs with dead bacteria, no cells could be found on the electrode surface.

### Microbial community analysis.

**FIG 5:**
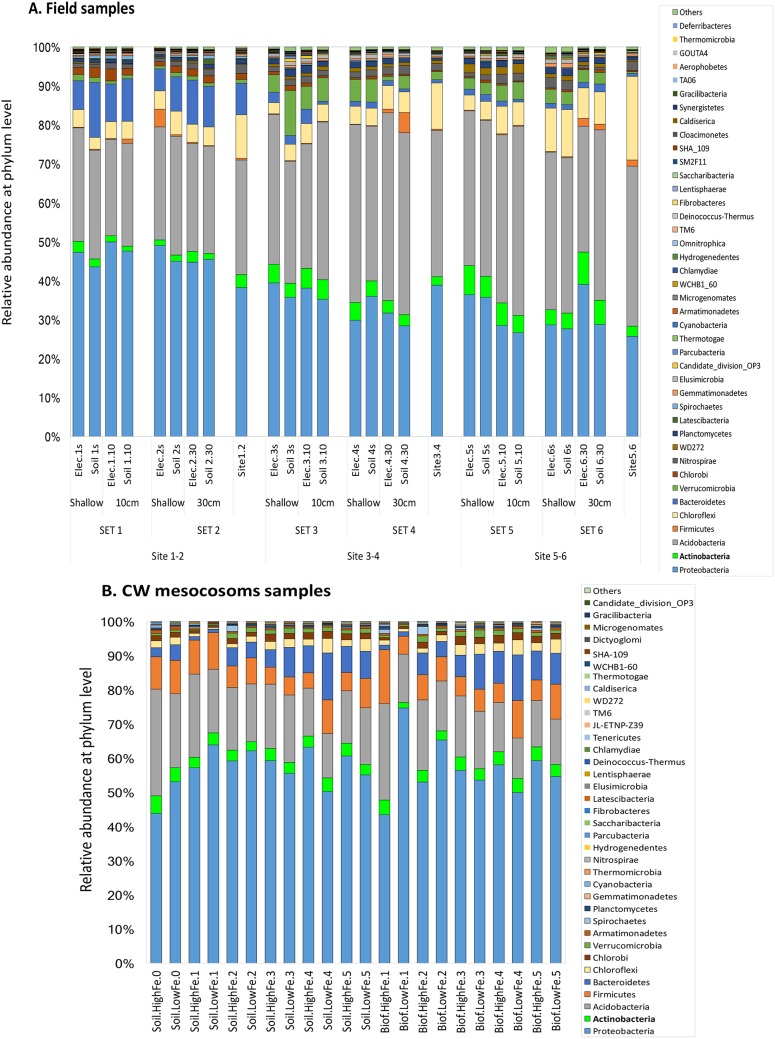
Microbial community composition, at the phylum level, of bacteria attached to electrodes and in soil samples from electrode pairs from three different field locations (A) and CW mesocosms (B). The *Actinobacteria* phylum (highlighted in green), to which *Acidimicrobiaceae* sp. strain A6 belongs, is among the most abundant phyla in all samples.

The microbial diversity at the phylum level found for the bacteria attached to the electrodes and in soil samples taken from the field, as well as the CW mesocosm studies (Fig. 5; see Table S1 in the supplemental material for data on archaea at the phylum level), showed that *Proteobacteria* and *Acidobacteria* represented, on average, more than 70% of the diversity found in all samples, followed in abundance by *Chloroflexi* and *Bacteroidetes* in the field study and by *Firmicutes* and *Bacteroidetes* in the CW mesocosm study. These highly abundant groups constituted more than 80% of the population found in all samples. All of these phyla are commonly found in soil and in bioelectrochemical systems, due to their electrode-reducing ability (12, 13, 32, 33); therefore, they are common subjects of study. *Actinobacteria*, the phylum to which *Acidimicrobiaceae* sp. strain A6 belongs, represented as little as 2, 4, 5, and 3.5% of the relative abundance found at the three different sites and in CW mesocosms. Still, *Actinobacteria* ranked in the 5 most abundant phyla found at each field site and in the CW mesocosms and was in the third position for some samples of bacteria attached to the electrode from the field study (Fig. 5).

### (i) Field experiment microbial community analysis.

The *Actinobacteria* phylum contains *Acidimicrobiaceae* sp. strain A6, which was described as an unidentified *Acidimicrobiales* strain at the genus level in samples from the field sites because its 16S rRNA gene sequence was not available in public databases at the time of the field study. The operational taxonomic unit (OTU) annotated as unidentified *Acidimicrobiales* had ≥97% sequence identity with A6, thus confirming the presence of this Feammox bacterium in our samples. A total of 316 genera were annotated in the microbial community analysis; however, between 51% (sites 1 and 2) and 69% (sites 5 and 6) of the OTUs could not be classified at this level and were added to the “other” category. Among the 100 most abundant genera, the unidentified *Acidimicrobiales* ranked 56th (Fig. S4). The genera with the greatest relative abundance at sites 1 and 2, characterized by waterlogged conditions, were *Sideroxydans* (*Proteobacteria*), an Fe(II) oxidizer (34), and *Geothrix* (*Acidobacteria*), a known ERB (35). At sites 3 and 4 and sites 5 and 6, the most abundant genera were *Bryobacter* (*Acidobacteria*), an aerobic heterotroph, “*Candidatus* Solibacter” (*Acidobacteria*), *Acidibacter* (Proteobacteria), an FeRB, the autotroph *Acidothermus* (*Actinobacteria*), and *Sorangium* (*Proteobacteria*). Other Fe-cycling bacteria found among the 100 most abundant genera were *Acidiferrobacter*, *Anaeromyxobacter*, *Ferritrophicum*, *Geobacter*, *Gallionella*, *Desulfobulbus*, and *Georgfuchsia* (all from the *Proteobacteria* phylum).

### (ii) CW mesocosm microbial community analysis.

A6 was identified in the CW sediments and among the bacteria attached to the electrodes, and A6 ranked 89th, on average, among the top 300 genera for all CW mesocosm samples (bacteria from soil and electrodes). Unclassified OTUs constituted 26.3 to 51.6% of the total sequences for CW soil samples and 17.4 to 49.5% of the total sequences for samples of bacteria attached to the electrodes. The two genera with the greatest relative abundance in the CW mesocosms for all samples (bacteria from soil and electrodes) were *Thiomonas* and *Burkholderia* (both from the *Proteobacteria* phylum). The third most abundant genus in the CW soil samples was *Telmatobacter* (*Acidobacteri*a), a group of anaerobes and chemo-organotrophs. In contrast, the third most abundant genus in the samples of bacteria attached to the electrodes was *Geobacter* (*Proteobacteria*).

The relative abundance of A6 in all CW samples was about 0.1 to 12.8% of *Geobacter* spp., which can be enriched on the anodes (3). Although A6 was not as abundant as *Geobacter* spp. in the CW mesocosms (Fig. S5), its population was still enriched on the anodes, compared to the surrounding sediments (Fig. 3). In addition to *Geobacter*, 10 other genera that include known electrogenic bacteria species were detected among the top 100 genera in all CW mesocosm samples, including *Geothrix*, *Desulfobulbus*, *Desulfovibrio*, *Pseudomonas*, *Clostridium* (2), *Desulfotomaculum* (36), *Enterobacter* (37), *Bacillus*, *Rhizomicrobium* (38), and *Anaeromyxobacter* (32). Most of the aforementioned electrogenic bacteria were more abundant on the electrodes than in the nearby soil samples (paired two-sample *t* test, *P* < 0.05) (Table S2). In fact, the electrogenic bacteria formed substantial portions of the microbial communities in the CW mesocosm samples, representing 3.0 to 8.5% of the total sequences for CW soil samples and 4.5 to 14.4% for bacteria attached to CW electrodes. The electrogenic bacteria that were enriched on electrodes, compared to the nearby soil, were all much more abundant than A6, resulting in a lower relative abundance of A6 on the electrodes, compared to the nearby soil (Fig. S5), even though the absolute numbers of A6 on the electrodes were higher than those in the soil (Fig. 3).

### Current pulse after injection of *Acidimicrobiaceae* sp. strain A6 enrichment culture into CWs.

**FIG 6:**
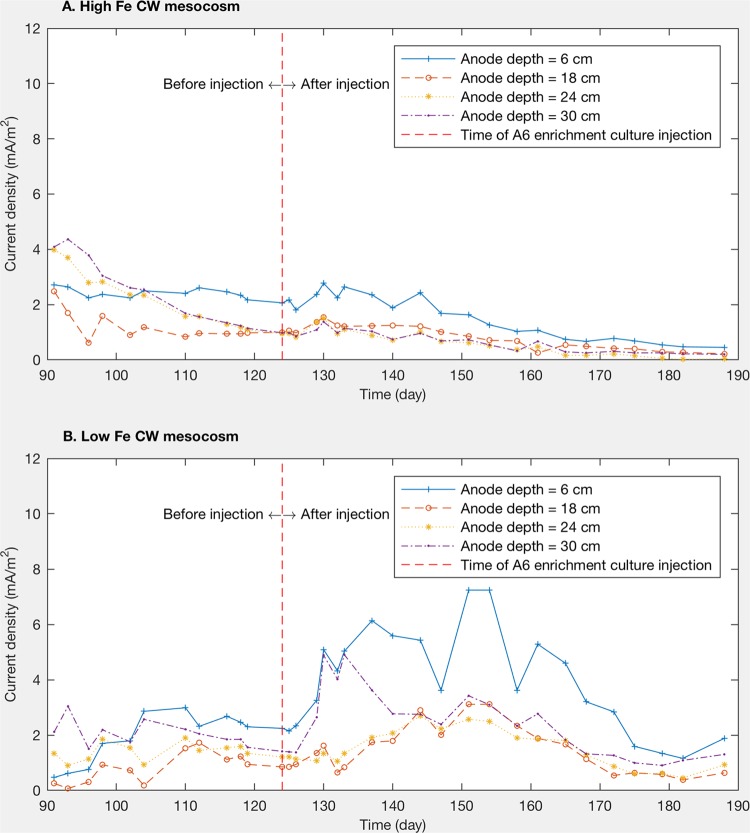
*I_d_* profiles for the high-Fe CW mesocosm (A) and the low-Fe CW mesocosm (B) 1 month before and 3 months after the injection of the A6 enrichment culture. Because of the ORP development, the second electrodes (at a depth of 12 cm) had the highest redox potential when the A6 enrichment culture was injected. Therefore, the electrodes at a depth of 12 cm in both CW mesocosms were connected as cathodes. The currents were measured for electrode pairs with different anode depths.

Although the high-Fe and low-Fe CW mesocosms had similar current profiles before the injection of the A6 enrichment culture, the current profiles right after the injection showed a noticeable difference (Fig. 6). The electrical current between electrode pairs in the low-Fe CW mesocosms increased 5 days following the A6 enrichment culture injection and then decreased to the previous level after 50 days. However, currents between electrode pairs in the high-Fe CW mesocosms remained within a similar range or increased only slightly. The different responses indicate that more electrons were transferred through the electrode pairs in the low-Fe mesocosm than in the high-Fe mesocosm, as the same amounts of A6 were introduced into the two mesocosms.

## DISCUSSION

### *Acidimicrobiaceae* sp. strain A6 colonization and electron transfer to electrodes.

The numbers of A6 quantified on the electrodes, in contrast to the surrounding soils, is consistent with the hypothesis that this strain is able to colonize electrodes. Furthermore, the higher bacterial counts on the deeper electrodes in the laboratory setup (Fig. 2) and on the electrodes in the more reduced soil in the CWs (Fig. 3) and the production of current by the pure culture in the MECs (Fig. 4) confirm the affinity of A6 for the electrode working as an anode. This means that A6 is able to use the electrode as an electron acceptor alternative to Fe(III), thus promoting biomass enhancement, compared to the surrounding soil (Fig. 1).

In the CWs, the consistent trend of A6 enrichment on electrodes was found on the anodes but not always on the electrodes that had been operated as cathodes for a limited period (electrodes 1 and 2 in the CW). Similarly, in the soil columns that were kept saturated throughout the incubation, in which there was no polarity switch in the electrodes, greater enrichment was found on the anode. In addition, no A6 enrichment was found on unconnected graphite plates placed in the field or in the laboratory soil columns.

It is interesting to note that injection of A6 into the low-Fe CW resulted in a current pulse, while only a slight pulse was observed in the high-Fe CW. This suggests that, when little bioavailable Fe was present, A6 showed a more immediate affinity to colonize and to transfer electrons to the electrodes. Although the field experiments and the laboratory soil columns were not designed to control Fe availability, we found that, in general, there was greater A6 enrichment at sites with lower soil Fe(III) levels (Table S3), which is consistent with the greater current production in CWs under conditions with lower levels of bioavailable Fe. It should be noted that the samples for DNA extraction were obtained almost 4 months after the injection of A6 and 3 months after the current pulse disappeared in the low-Fe mesocosm. Therefore, microbial analysis results and A6 numbers discussed above may not properly capture the microbial community at the time of the current pulse. The decrease in the current about 50 days after the injection indicates that A6 numbers and activity on the electrodes in the low-Fe CW decreased over time after the injection; therefore, A6 may have been more enriched on the electrodes when the current pulse occurred in the low-Fe CW mesocosm. However, since the DNA extraction requires destructive sampling, analyses of A6 populations during the operation of CW mesocosms are not available. Since A6 can use electron shuttles such as AQDS (10, 19) to enhance the Feammox process and since natural organic matter (NOM) may function as a mediator for transferring electrons to Fe(III) during anaerobic NH_4_^+^ oxidation (39), some electrons from the oxidation of NH_4_^+^, and hence the current measured, could have been transferred to the anode via an electron-shuttling compound (NOM in the field experiments and CWs and AQDS in the MECs), without the need for direct colonization of the anode.

### *Acidimicrobiaceae* sp. strain A6 in MECs.

Because the A6 enrichment culture was injected and no microbial analysis was available for the period of the current pulse in the CW mesocosms, it is not certain that the current was produced by A6 itself. Therefore, MECs with pure A6 strain were used to clarify the ability of A6 to produce current in the absence of other microorganisms. The results showed that pure A6 culture produced current when run in MECs with an applied voltage of 0.7 V and constant shaking, confirming its ability to use the anode as an electron acceptor in the absence of Fe(III). However, we have not been successful in maintaining a viable pure A6 culture for long periods (10), which could explain the decrease in current production in MECs.

It has been reported that biological NH_4_^+^ removal in MFCs is more efficient than NH_4_^+^ removal via an abiotic electrochemical pathway (40) and that NH_4_^+^ removal can be enhanced by implementing the Feammox process in air-cathode MFCs (41). However, none of those studies identified the microorganisms that were responsible for the NH_4_^+^ removal. The results presented in this study, especially from the MECs with pure A6 inoculation, characterized the ability of A6 to colonize and to transfer electrons onto electrodes, which raises the possibility of applying A6 in bioelectrochemical systems for NH_4_^+^ treatment.

### Correlation of relative abundances of *Acidimicrobiaceae* sp. strain A6 and other bacteria.

A6 ranked 56th in relative abundance at the genus level in the field study soil and electrode samples and much lower (89th) in the CW soil and electrode samples, being outranked by other FeRB. It is not surprising that other FeRB, such as *Geobacter* spp., were more abundant than A6 in both the field and the CWs. This finding could be due to the fact that *Geobacter* is a heterotroph and thus has a much faster doubling time (5 h to less than 1 day, depending on the species), compared to lithoautotrophs like A6, which has a doubling time of 8 to 10 days (10).

**FIG 7:**
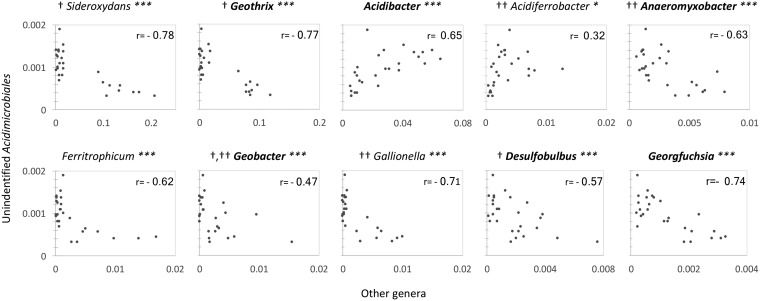
Correlations of the relative abundances of *Acidimicrobiaceae* sp. strain A6 (unidentified *Acidimicrobiales*) and Fe-cycling bacteria attached to the electrodes and in soil samples (*n* = 27). Fe-oxidizing bacteria are in italics, and Fe-reducing bacteria are in bold italics. †, anode colonizer (24, 32, 35, 53); ††, cathode colonizer (32). ***, *P* < 0.01; *, *P* < 0.1.

**FIG 8:**
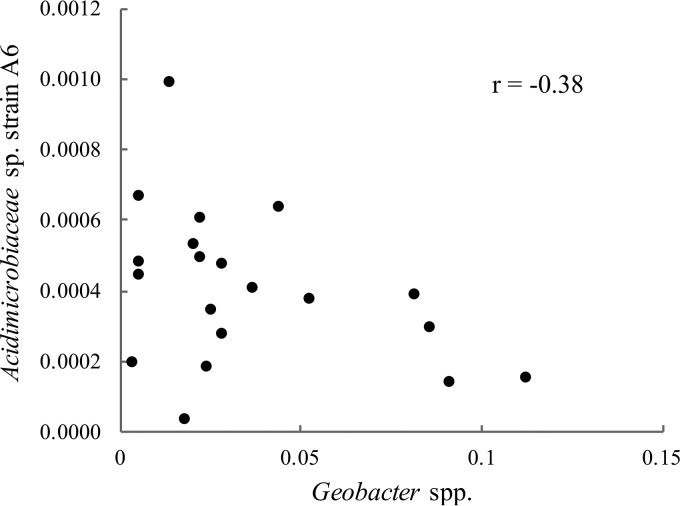
Correlation of the relative abundances of *Acidimicrobiaceae* sp. strain A6 and *Geobacter* spp. in the CW sediments and among bacteria attached to the electrodes (*P* < 0.1).

The relative abundance of A6 in field soil and electrode samples showed negative correlations with that of other FeRB (Fig. 7). This was not the case for the relative abundance of A6 in comparison with non-metal-reducing bacteria in field soil and electrode samples, for which positive or no correlations were observed (*r* ≈ 0.0) (Fig. S6). When only the relative abundances of bacteria attached to the electrodes were analyzed for correlations, the correlation between A6 and *Collimonas* shifted from slightly negative (*r* = −0.02) to positive (*r* = 0.45; *P* > 0.1). For all other genera, the trends of the correlations were maintained when all of the data (bacteria from soil and electrodes) were pooled for analysis or separated according to bacteria on the electrodes or in soil samples only. This indicates, that in soils and on the electrodes, the presence of A6 was negatively affected by most other Fe-cycling bacteria found in our samples. When the relative abundance of *Geobacter* was correlated with that of other Fe-cycling bacteria, it showed positive correlations with all except *Acidibacter* and *Acidiferrobacter.* Similarly, negative correlations between A6 and *Geobacter* spp. (*r* = −0.38) in the sediments and on the electrodes of the CWs were observed (Fig. 8). These findings reveal the need for further research to elucidate what drives these correlations and whether they may indicate competition for the electron acceptor (iron or electrode) between A6 and other FeRB.

The results from this study show that *Acidimicrobiaceae* sp. strain A6, which is an iron reducer, is capable of colonizing electrodes in the field, in soil columns in the laboratory, and in CW mesocosms. Under controlled conditions, A6 was enriched on the anode, although in some cases A6 was also detected in smaller numbers on the cathode. The enrichment of A6 on the anode appears to be linked to the production of current in mixed systems like CWs. Furthermore, current production measured in MECs inoculated with pure A6 points to A6 being a new ERB capable of producing current in these systems. Thus, *Acidimicrobiaceae* sp. strain A6 is a novel anaerobic lithoautotroph from the *Actinobacteria* phylum that is capable of using electrodes as terminal electron acceptors. Altogether, this work expands the knowledge regarding the diversity of electrogenic microorganisms beyond the commonly studied groups and opens the possibility for applications of these bacteria in MFC and MEC systems. However, further research is needed to elucidate what drives the different interactions between A6 and other FeRB and ERB, in order to optimize the applications of A6 in bioelectrochemical systems.

## MATERIALS AND METHODS

In this study, electrodes were installed in multiple locations in a forested riparian wetland and in laboratory CW mesocosms, to investigate A6 as a potential ERB. Results from electrodes deployed in the field provide insights regarding the natural and electrode-enhanced growth conditions for A6, whereas electrodes deployed in the laboratory soil columns and the CW mesocosms provide controlled conditions to better understand the field findings. Finally, MECs inoculated with pure A6 culture provide information regarding the electrogenicity of A6.

### Field electrode construction and setup.

**FIG 9:**
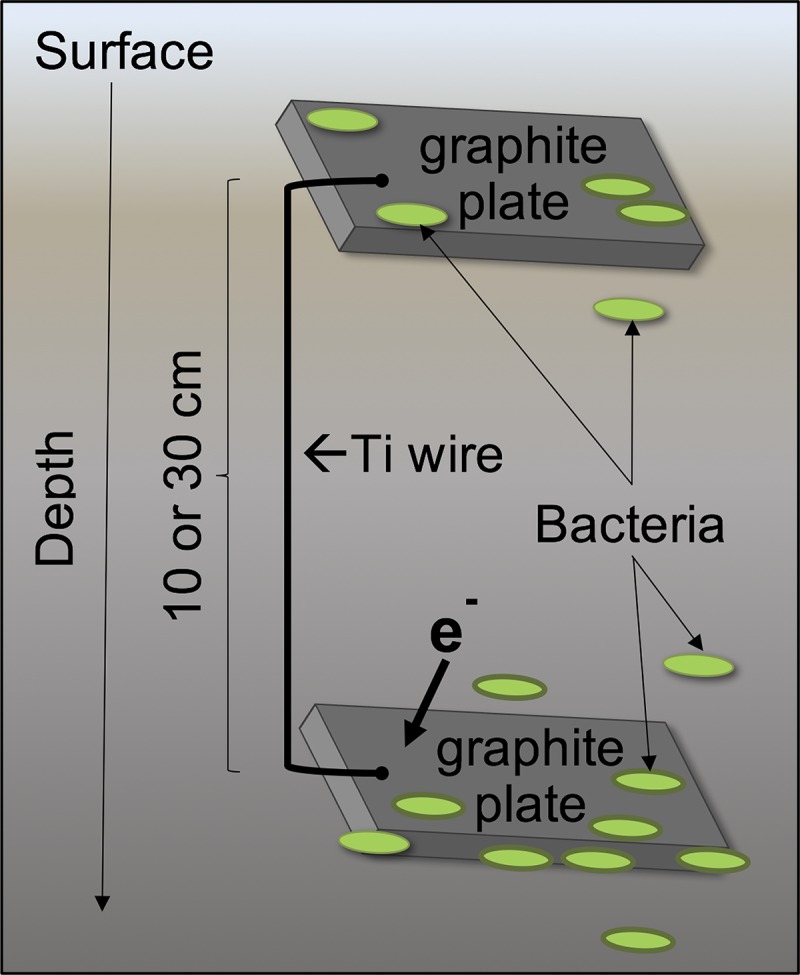
Schematic of an electrode pair.

Electrodes consisted of graphite plates (7.5 cm [length] by 2.5 cm [width] by 0.32 cm [height]), with a surface area per face of 18.75 cm^2^ (grade GM-10; GraphiteStore.com Inc.). The plates were polished using sandpaper (grit type 400), sonicated to remove debris, cleaned by overnight soaking in 1 N HCl, and rinsed three times in distilled water (41). Each electrode set was connected by a titanium wire cleaned with sandpaper (ultra-corrosion-resistant Ti wire, 0.08 cm in diameter; McMaster-Carr code 90455k32); the wire was inserted through two 0.08-cm holes drilled in each graphite plate to ensure a tight connection between the wire and the graphite plates, to allow for low contact resistance of <0.5 Ω. The Ti wire was long enough to allow 10 or 30 cm of separation between the graphite plates (Fig. 9).

Two pairs of electrodes were placed at three different sites. Each pair consisted of a shallow electrode placed no deeper than 5 cm into the soil, connected to another electrode with either 10 cm or 30 cm of separation, i.e., a total of 6 sets (Table 2), in a temperate forested riparian wetland located in the Assunpink Wildlife Management Area (WMA) in New Jersey, USA. This is the location where the Feammox reaction was first discovered (8); later, the Feammox bacterial strain A6 was identified in samples from this site (7) and isolated (10). Detailed physicochemical characteristics of the soil were described in previous studies (8, 42). Electrode sets 1 and 2 were placed in a fully flooded location, and sets 3 to 6 were placed in a wet but unsaturated location. The electrode sets were left in the field for 52 days, between 13 June 2016 and 3 August 2016.

**TABLE 2:** Description of bacteria attached to electrodes, on control graphite plates, and in soil samples

Set category	Electrode set/soil	Electrode/soil sample	Sample name
Electrode sets placed at Assunpink WMA for 52 days	Set 1	Shallow electrode 1	Elec. 1s
			Soil 1s
		10-cm-deep electrode 1	Elec. 1.10
			Soil 1.10
	Set 2	Shallow electrode 2	Elec. 2s
			Soil 2s
		30-cm-deep electrode 2	Elec. 2.30
			Soil 2.30
	Sites 1 and 2	Soil from sites 1 and 2	Soil 1-2
	Set 3	Shallow electrode 3	Elec. 3s
			Soil 3s
		10-cm-deep electrode 3	Elec. 3.10
			Soil 3.10
	Set 4	Shallow electrode 4	Elec. 4s
			Soil 4s
		30-cm-deep electrode 4	Elec. 4.30
			Soil 4.30
	Sites 3 and 4	Soil from sites 3 and 4	Soil 3-4
	Set 5	Shallow electrode 5	Elec. 5s
			Soil 5s
		10-cm-deep electrode 5	Elec. 5.10
			Soil 5.10
	Set 6	Shallow electrode 6	Elec. 6s
			Soil 6s
		30-cm-deep electrode 6	Elec. 6.30
			Soil 6.30
	Sites 5 and 6	Soil from sites 5 and 6	Soil 5-6
Electrode and control sets placed at Assunpink WMA for 32 days	Set 7	Shallow electrode 7	Elec. 7s
			Soil 7s
		10-cm-deep electrode 7	Elec. 7.10
			Soil 7.10
	Set 8	Shallow electrode 8	Elec. 8s
			Soil 8s
		30-cm-deep electrode 8	Elec. 8.30
			Soil 8.30
	Control	10-cm-deep control plate	Plate C.10
			Soil C.10
		30-cm-deep control plate	Plate C.30
			Soil C.30
	Sites 7 and 8	Soil from sites 7 and 8	Soil 7-8
Electrode and control sets placed in laboratory soil cores for 52 days	Set 9	Shallow electrode 9	Elec. 9s
			Soil 9s
		30-cm-deep electrode 9	Elec. 9.30
			Soil 9.30
	Control	Shallow control plate	Plate C.s
			Soil C.s
		30-cm-deep control plate	Plate C.30
			Soil C.30

A separate set of experiments were carried out as controls. First, electrode pairs with configurations identical to those described above were placed in triplicate in the Assunpink WMA, triplicates of unconnected graphite plates (10 cm and 30 cm deep) were placed as controls, and all of them were left in the field for 32 days (Table 2). Second, soil cores from the same location in the Assunpink WMA were brought to the laboratory to set up an identical experiment in which the water level was maintained at saturation throughout the experiment by the addition of deionized water, to avoid possible redox fluctuations and changes in polarity due to drying of the soils (Table 2).

### Field electrode recovery and sampling.

After 52 days of deployment in the field for the first group of electrodes and 32 days of deployment for the second group, the electrode pairs were recovered by digging them out of the soil, and each electrode was placed individually in a sealed bag. All of the electrodes were surrounded by soil. Furthermore, a soil sample was taken from each site, from a depth of about 20 cm, and placed in a sealed bag. All samples were transported to the laboratory within 2 h and immediately stored at 4°C until processing for analysis. The same recovery procedure was applied to the electrodes placed in the laboratory soil cores, which were dismantled after 52 days.

The samples obtained from the electrodes and soil are enumerated in Table 2. To analyze the biomass attached to the electrodes, the following procedure was used. First, loosely bound soil was removed by gently shaking the electrode. Second, duplicate samples were taken from the soil layer (<2 mm thick) still surrounding the electrode. Third, duplicate samples of the biomass formed on the electrode surface together with some graphite were removed using a sterile cutting blade (see Table S4A in the supplemental material for details). DNA was extracted from all samples and then used for determination, quantification, and comparison of the microbial composition (the latter only for the first group).

### CW mesocosm and electrode configuration.

**FIG 10:**
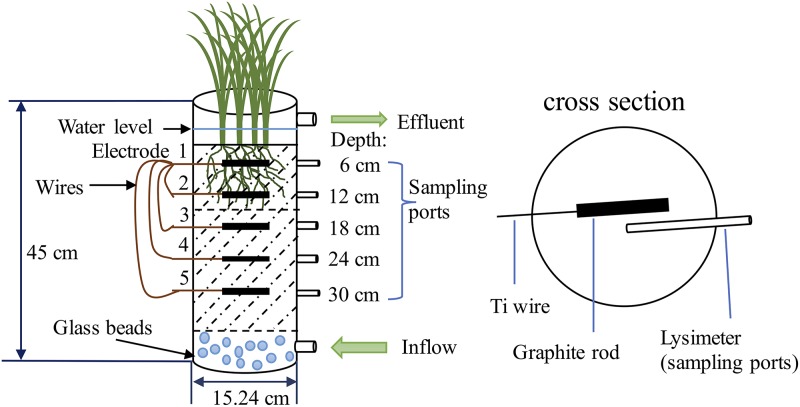
Schematic of a CW mesocosm and electrode configuration.

CWs were set up and operated with two different Fe levels in their sediments, to determine how Fe levels affected the removal of NH_4_^+^ via Feammox after augmentation with A6. The CW with higher Fe levels had higher A6 counts and greater NH_4_^+^ removal (43). For the purposes of this study and to establish well-controlled conditions to gain further insights into the electrogenesis of A6, electrode pairs were installed in the two CW mesocosms. The dimensions of the mesocosms and the design of sampling ports are shown in Fig. 10. Briefly, the CWs were designed as continuous up-flow mesocosms with water surface above the sediment. Synthesized Fe_2_O_3_·0.5H_2_O (2-line ferrihydrite) was added to one of the CW mesocosms to elevate its initial Fe(III) level. After the substrates were blended thoroughly, Fe(III) levels were measured for the high-Fe CW mesocosm [2.9 g Fe(III)/kg dry soil] and low-Fe CW mesocosm [1.7 g Fe(III)/kg dry soil] substrates. The CW substrates were inoculated with A6 by adding 250 ml of an A6 enrichment culture (10^9^ to 10^10^ CFU/g sludge; 70% A6 in biomass) to the CW substrate of each column. The substrate was then thoroughly mixed prior to loading into the mesocosm columns. The CWs were planted with *Scirpus acutus* (bulrush) and were operated in a growth chamber (Environmental Growth Chambers) simulating the summer climate of New Jersey (Table S5), as described previously (43).

To investigate whether A6 could colonize the electrodes and transfer electrons onto anodes, five graphite electrodes were installed in each mesocosm at the same depths as the five sampling ports. The electrodes were made of rectangular graphite rods (10.16 cm [length] by 1.28 cm [width] by 1.28 cm [height]). The preparation of the electrodes and wires was the same as described above. Each of the four electrodes that had been placed in the more reduced zones (anode) was then connected, via a cleaned titanium wire, to the electrode that had been placed in the most oxidized zone (cathode) of the CW, as shown in Fig. 10.

### CW mesocosm operation.

Details of the CW mesocosm operation were given in a previous report (43). After 4 months of operation, when the mesocosm had become more reduced and plants were fully established, another A6 enrichment culture (around 10^7^ A6 counts/ml of culture; 250 ml per CW mesocosm) was injected from the bottom into each CW on day 124, to ascertain the colonization of A6 in the mesocosms. This procedure also allowed determination of whether a spike in A6 numbers would result in an increase in electrical current. The nutrient solution contained acetic acid to deplete any DO (DO levels of <0.1 ppm) entering the CWs.

### CW mesocosm sampling and dismantlement.

The ORPs in the CWs were measured by taking water samples from the sampling ports and collecting effluents from the top of the CWs. At the end of the experiment, the CW mesocosms were dismantled and soil samples were taken for analysis of microbial communities. Soil sampling was described previously (43). Electrodes installed in the CW mesocosms were removed carefully, and attached bacterial samples from the electrodes were obtained using the same procedure as described above (see Table S4B in the supplemental material for details). Since soil and electrode sampling requires sacrifice of the mesocosms, no soil or electrode samples were collected during the operation of the CW mesocosms. Samples obtained from the CW mesocosm sediments and electrodes are enumerated in Table 3.

**TABLE 3:** Description of bacteria attached to electrodes and in soil samples taken from CW mesocosms

Depth (cm)	Electrode	Mesocosm	Sample name
0–3	Top sediment	High-Fe CW	Soil High Fe.0
		Low-Fe CW	Soil Low Fe.0
6–9	Electrode 1	High-Fe CW	Soil High Fe.1
			Elec. High Fe.1
		Low-Fe CW	Soil Low Fe.1
			Elec. Low Fe.1
12–15	Electrode 2	High-Fe CW	Soil High Fe.2
			Elec. High Fe.2
		Low-Fe CW	Soil Low Fe.2
			Elec. Low Fe.2
18–21	Electrode 3	High-Fe CW	Soil High Fe.3
			Elec. High Fe.3
		Low-Fe CW	Soil Low Fe.3
			Elec. Low Fe.3
24–27	Electrode 4	High-Fe CW	Soil High Fe.4
			Elec. High Fe.4
		Low-Fe CW	Soil Low Fe.4
			Elec. Low Fe.4
30–33	Electrode 5	High-Fe CW	Soil High Fe.5
			Elec. High Fe.5
		Low-Fe CW	Soil Low Fe.5
			Elec. Low Fe.5

### MEC construction, setup, and operation.

MECs were constructed and run in parallel, as described previously (41). Five MECs were run with an applied voltage of 0.7 ; two were inoculated with pure live A6 culture, two were inoculated with A6 culture and then autoclaved (dead A6), and one contained only sterile Feammox medium. The Feammox medium consisted of 5 mM NH_4_Cl, 0.24 mM NaHCO_3_, 0.71 mM KHCO_3_, 0.052 mM KH_2_PO_4_, 0.41 mM MgSO_4_·7H_2_O, 0.54 mM CaCl_2_, 0.1 μl/liter vitamin supplement (ATCC), and trace element solution; the latter solution was described previously (9). MECs were operated for 2 months at room temperature, on a shaker plate. The first 2 weeks were for acclimation, after which the Feammox medium was replaced with fresh medium. During the operation, current (*I*) was measured using a multimeter. Initial and final NH_4_^+^ concentrations were measured after medium replacement and at the end of the operation. A Dionex ICS3000 ion chromatographic system, using a CS-16 column with a CS-16 guard column and a CERS 500 (4-mm) suppressor, was used for the NH_4_^+^ analyses. DNA was extracted from all MECs and then used for quantification of A6 and total bacteria, as described below.

### DNA extraction, *Acidimicrobiaceae* sp. strain A6 quantification, and microbial community analysis.

Total genomic DNA was extracted from attached bacteria samples obtained in duplicate from a half face or full face of each electrode deployed in the field, except for the deep electrode of set 6, which was only partially recovered and for which only one sample could be obtained from all faces. Since many electrodes in the CWs had much lower masses of attached bacteria than the filed electrodes, only one sample was recovered for DNA extraction from the CW electrodes. DNA was also extracted from each soil sample, as described above (Table S4). For MECs, DNA was extracted from the liquid culture. Extractions were performed using the FastDNA spin kit for soil (MP Biomedicals), according to the manufacturer’s instructions, with an additional first step for MECs in which the bacteria were concentrated by centrifuging the liquid medium for 10 min at 14,000 relative centrifugal force (RCF), equivalent to 12,750 revolutions per minute (rpm); the pellet was resuspended in 500 µl of the supernatant and used as the initial substrate for extraction. Total DNA was eluted in 100 µl of sterile water, and DNA concentrations were measured using Qubit 2.0 (Invitrogen). All DNA samples were preserved at −20°C until further analysis. Since at this point neither the mechanism by which A6 oxidizes NH_4_^+^ nor its functional gene has been identified, we used 16S rRNA gene sequencing for two tasks, (i) for quantification of A6 using specific primers designed using the genomic sequence of A6 (10) and (ii) for taxonomic annotation.

Quantification of bacteria was carried out via qPCR using the Applied Biosystems StepOnePlus real-time PCR system. A6 quantification was performed by amplifying a section of the 16S rRNA gene between the V1 and V4 variable regions using primer set 33F/232R (33F, 5′-GGCGGCGTGCTTAACACAT-3′; 232R, 5′-GAGCCCGTCCCAGAGTGATA-3′). *Geobacter* spp. (electrogenic bacteria known for the ability to colonize electrodes) were quantified by amplifying a region of the 16S rRNA gene using primer set 561F/825R (561F, 5′-GCGTGTAGGCGGTTTCTTAA-3′; 825R, 5′ATCTACGGATTTCACTCCTACA-3′). For MECs, A6 was amplified using the primer set acm342F/439R, following the protocol described previously (7).

Each qPCR mixture (20 μl) was composed of 10 μl of SYBR Premix *Ex Taq* II 2× (TaKaRa, Japan), 0.8 μl of each forward and reverse primer at 10 μM, and the DNA template. Thermal cycling was initiated with 30 s at 95°C, followed by 40 cycles with different times and temperatures depending on the amplicon being generated, and it ended with a melting curve analysis for the SYBR green assay, which was used to distinguish the targeted PCR product from the nontargeted PCR products. For A6 amplification, the cycling consisted of 10 s at 95°C, 15 s of annealing at 59°C, and 15 s at 72°C. For total bacterial quantification, each cycle consisted of 5 s at 94°C, 30 s at 55°C, and 30 s at 70°C. Each qPCR assay was run in duplicate or triplicate for each sample and included negative controls and a standard curve; the latter consisted of serial dilutions of known numbers of copies of DNA. Finally, the results were converted into copies of DNA per square meter by dividing the total gene copies obtained from the qPCR by the surface area of sediments for soil samples or by the surface area of the electrode for the samples of bacteria attached to electrodes.

In order to determine the microbial community composition and abundance in the sediments (field and CW) and to compare them with those formed on the electrodes, sequencing and microbial community analysis were performed by Novogene (Beijing, China), as follows. From total genomic DNA, the V4 variable region of the 16S rRNA gene was amplified using the primer set 515F/806R (515 F, 5′-GTGCCAGCMGCCGCGGTAA-3′; 806R, 5′-GGACTACHVGGGTWTCTAAT-3′), with a barcode, following the method described previously (44). All PCRs were carried out with Phusion high-fidelity PCR master mix (New England Biolabs). PCR product quantification and qualification were performed by electrophoresis on 2% agarose gels. The resulting amplicons were pooled, purified, and quantified. Sequencing libraries were generated using a TruSeq DNA PCR-free sample preparation kit (Illumina), following the manufacturer’s protocol, and index codes were added. The library quality was assessed with a Qubit 2.0 fluorometer (Thermo Scientific) and Agilent Bioanalyzer 2100 system. Finally, sequencing was performed on an Illumina HiSeq 2500 platform, and 250-bp paired-end reads were generated.

Paired-end reads were assembled by using FLASH v1.2.7 (45). Raw reads were processed according to the QIIME v1.7.0 quality control process (46), and chimeric sequences were filtered out using the UCHIME algorithm (47). For all samples (field and CW), at least 25,000 sequences were obtained. These sequences were clustered into OTUs using Uparse v7.0.1001 (48). Sequences with ≥97% similarity were assigned to the same OTU. A total of 3,206 OTUs were produced across all field samples and 2,870 OTUs were produced across all CW mesocosm samples, with ranges of 1,422 to 1,794 OTUs per field sample and 674 to 1,481 OTUs per mesocosm sample. A representative sequence for each OTU was screened for taxonomic annotation with the RDP Classifier (49, 50), using the Greengene database (51), at a confidence threshold of at least 80% for all OTUs. For CW samples, the OTUs were screened for taxonomic annotation by applying the BLASTn algorithm against the 2016 NCBI 16S rRNA sequences for bacteria and archaea, at an E value of 1*e*^−5^. The 16S rRNA gene sequence of A6 was included with the databases for annotation at the family and genus levels for the 100 and 300 most abundant OTUs for the CW samples. Finally, samples were standardized using the lowest sequence number obtained from all samples, so that the same numbers of sequences were used to calculate the relative abundances of OTUs.

### Soil surface area analysis.

Nitrogen sorption was used to determine the surface area of the soil samples taken from the field (Table 2) and the CWs (Table 3). Prior to the analysis, samples were oven dried at 56°C until the mass stabilized. Subsequently, the samples were degassed at 60°C and 0.1 mm Hg, using a Micromeritics Smart VacPrep system (Micromeritics, Norcross, GA, USA). The nitrogen sorption measurements were conducted using a Micromeritics 3Flex analyzer, using the Brunauer-Emmett-Teller (BET) method to calculate the surface area of the soil. The measurements obtained were used to normalize the bacterial count data by surface area.

### Analytical methods.

The iron concentrations in the sediments were analyzed using the ferrozine method (52). Briefly, a 0.5-g sediment sample was added to 9.5 ml of 0.5 M HCl and shaken for 24 h at room temperature to extract Fe(II). In total, 60 μl of 6.25 M NH_2_OH·HCl was added to 3 ml of extraction solution, and the mixture was shaken for 24 h at room temperature to reduce Fe(III) to Fe(II). For the chromogenic reaction, 60 μl of extraction solution was added to 3 ml 1 g/liter ferrozine solution (pH 7.0), and the reaction was allowed to proceed for 30 min. The concentrations of Fe(II) and total Fe were measured by reading the absorbance at 562 nm using a Spectronic Genesys 2 instrument, and the Fe(III) concentration was calculated from the difference. ORPs at different depths of CW mesocosms were measured using probes from Thermo Scientific. During the operation of CW mesocosms, electrode pairs were connected using only wires, whereas 1,000-Ω resistors were connected in the circuit for voltage measurements. The voltages between electrode pairs in the CW mesocosms were measured using a multimeter, and currents were calculated accordingly.

### Statistical analysis.

The Welch *t* test was used to determine whether there were statistical differences in bacterial counts per surface area between electrode and soil samples in the field and CW mesocosm experiments. Paired two-sample *t* tests were conducted to determine whether there were statistical differences in relative abundance for electrode/soil pairs in the CW mesocosms.

## Supplementary Material

Supplemental file 1
