# Supplementary material for: Electrode Colonization by the Feammox Bacterium Acidimicrobiaceae sp. Strain A6
Source: Appl Environ Microbiol. 2018 Nov 30;84(24):e02029-18. doi: 10.1128/AEM.02029-18 (PMC6275345; doi:10.1128/AEM.02029-18)
Supplement: Supplemental file 1 [file zam024188888s1.pdf]

## Supplemental Materials

# Electrode Colonization by the Feammox Bacterium *Acidimicrobiaceae* sp. Strain A6

Melany Ruiz-Urigüen, Weitao Shuai, Peter R. Jaffé

**Table S1. A)** Relative abundance of Archaea at phylum level in field samples

| Site | Set | Sample name | Phylum name | | | | | |
|---|---|---|---|---|---|---|---|---|
| | | | MEG | Crenarchaeota | Euryarchaeota | MCG | Thaumarchaeota | Woesearchaeota DHVEG-6 |
| Site 1-2 | 1 | Elec. 1s | 0 | 0 | 0 | $8.00\times10^{-5}$ | $4.00\times10^{-4}$ | 0 |
| | | Soil 1s | 0 | 0 | 0 | $4.00\times10^{-5}$ | 0 | 0 |
| | | Elec. 1.10 | 0 | 0 | 0 | $4.00\times10^{-5}$ | 0 | 0 |
| | | Soil 1.10 | 0 | 0 | 0 | 0 | 0 | 0 |
| | 2 | Elec. 2s | 0 | 0 | 0 | 0 | 0 | 0 |
| | | Soil 2s | 0 | 0 | 0 | 0 | 0 | 0 |
| | | Elec. 2.30 | 0 | 0 | 0 | 0 | $8.00\times10^{-5}$ | 0 |
| | | Soil 2.30 | 0 | 0 | 0 | 0 | 0 | 0 |
| | | Soil 1-2 | 0 | 0 | 0 | $8.00\times10^{-5}$ | $4.00\times10^{-5}$ | 0 |
| Site 3-4 | 3 | Elec. 3s | 0 | 0 | $8.00\times10^{-5}$ | 0 | $4.80\times10^{-4}$ | 0 |
| | | Soil 3s | $8.00\times10^{-5}$ | 0 | $8.00\times10^{-4}$ | $4.00\times10^{-5}$ | $2.76\times10^{-3}$ | 0 |
| | | Elec. 3.10 | $4.00\times10^{-5}$ | 0 | $4.00\times10^{-5}$ | $2.40\times10^{-4}$ | $2.60\times10^{-3}$ | $4.00\times10^{-5}$ |
| | | Soil 3.10 | $1.20\times10^{-4}$ | 0 | $2.24\times10^{-3}$ | 0 | $1.28\times10^{-3}$ | 0 |
| | 4 | Elec.4s | $4.00\times10^{-5}$ | 0 | $8.00\times10^{-4}$ | $1.60\times10^{-4}$ | $7.60\times10^{-4}$ | 0 |
| | | Soil 4s | $1.20\times10^{-4}$ | 0 | $1.04\times10^{-3}$ | 0 | $5.60\times10^{-4}$ | 0 |
| | | Elec. 4.30 | 0 | 0 | $4.00\times10^{-5}$ | $4.00\times10^{-5}$ | 0 | 0 |
| | | Soil 4.30 | $4.00\times10^{-5}$ | 0 | $6.00\times10^{-4}$ | $3.20\times10^{-4}$ | $1.08\times10^{-3}$ | $8.00\times10^{-5}$ |
| | | Soil 3-4 | 0 | $1.60\times10^{-4}$ | $2.00\times10^{-4}$ | $1.20\times10^{-4}$ | $1.44\times10^{-3}$ | $8.00\times10^{-5}$ |
| Site 5-6 | 5 | Elec. 5s | 0 | 0 | $2.00\times10^{-4}$ | $4.00\times10^{-5}$ | $7.20\times10^{-4}$ | 0 |
| | | Soil 5s | 0 | 0 | $2.00\times10^{-4}$ | 0 | $4.80\times10^{-4}$ | 0 |
| | | Elec. 5.10 | 0 | 0 | $3.20\times10^{-4}$ | $8.00\times10^{-5}$ | $5.56\times10^{-3}$ | 0 |
| | | Soil 5.10 | $4.00\times10^{-5}$ | 0 | $3.60\times10^{-4}$ | $8.00\times10^{-5}$ | $5.56\times10^{-3}$ | $4.00\times10^{-5}$ |
| | 6 | Elec. 6s | $4.00\times10^{-5}$ | $1.20\times10^{-4}$ | $6.40\times10^{-4}$ | $4.00\times10^{-5}$ | $5.72\times10^{-3}$ | $1.60\times10^{-4}$ |
| | | Soil 6s | 0 | $2.00\times10^{-4}$ | $5.20\times10^{-4}$ | $4.00\times10^{-5}$ | $5.20\times10^{-3}$ | $2.00\times10^{-4}$ |
| | | Elec. 6.30 | 0 | 0 | $3.20\times10^{-4}$ | 0 | $5.40\times10^{-3}$ | 0 |
| | | Soil 6.30 | 0 | $4.00\times10^{-5}$ | $4.40\times10^{-4}$ | $4.00\times10^{-5}$ | $3.56\times10^{-3}$ | $1.60\times10^{-4}$ |
| | | Soil 5-6 | 0 | 0 | $8.00\times10^{-5}$ | 0 | $1.20\times10^{-4}$ | 0 |

**Table S1. B)** Relative abundance of Archaea at phylum level in CW mesocosm samples

| **Depth (cm)** | **Sample name** | **Phylum name** | |
|---|---|---|---|
| | | **MCG** | **Thaumarchaeota** |
| **6** | Elec. High Fe.1 | 0 | 0 |
| | Soil High Fe.1 | 0 | 0 |
| | Elec. Low Fe.1 | 0 | 0 |
| | Soil Low Fe.1 | 0 | 0 |
| **12** | Elec. High Fe.2 | $1.47\times10^{-4}$ | 0 |
| | Soil High Fe.2 | $1.84\times10^{-5}$ | 0 |
| | Elec. Low Fe.2 | $1.84\times10^{-5}$ | $5.53\times10^{-5}$ |
| | Soil Low Fe.2 | $1.84\times10^{-5}$ | 0 |
| **18** | Elec. High Fe.3 | $9.22\times10^{-5}$ | $9.22\times10^{-5}$ |
| | Soil High Fe.3 | $7.37\times10^{-5}$ | $1.84\times10^{-5}$ |
| | Elec. Low Fe.3 | $7.37\times10^{-5}$ | $1.84\times10^{-5}$ |
| | Soil Low Fe.3 | $1.11\times10^{-4}$ | $1.84\times10^{-5}$ |
| **24** | Elec. HighFe.4 | $5.53\times10^{-5}$ | 0 |
| | Soil High Fe.4 | $5.53\times10^{-5}$ | 0 |
| | Elec. Low Fe.4 | $9.22\times10^{-5}$ | 0 |
| | Soil Low Fe.4 | $1.11\times10^{-4}$ | $3.69\times10^{-5}$ |
| **30** | Elec. High Fe.5 | $5.53\times10^{-5}$ | $1.84\times10^{-5}$ |
| | Soil High Fe.5 | $1.66\times10^{-4}$ | $7.37\times10^{-5}$ |
| | Elec. Low Fe.5 | 0 | 0 |
| | Soil Low Fe.5 | $7.37\times10^{-5}$ | 0 |

**Table S2.** Relative abundance of electrogenic bacteria detected in the CW mesocosm samples. Paired two sample t-test for means (two tail) were conducted for attached bacteria on the electrodes and their nearby soil samples.

| | ***Geobacter*** | | ***Geothrix*** | | ***Bacillus*** | |
|---|---|---|---|---|---|---|
| **Sample** | Soil | Electrodes | Soil | Electrodes | Soil | Electrodes |
| **HighFe.1** | $5.31\times10^{-3}$ | $2.39\times10^{-2}$ | $4.85\times10^{-3}$ | $5.92\times10^{-3}$ | $2.65\times10^{-2}$ | $3.38\times10^{-2}$ |
| **LowFe.1** | $3.32\times10^{-3}$ | $1.81\times10^{-2}$ | $4.12\times10^{-3}$ | $4.22\times10^{-3}$ | $2.86\times10^{-3}$ | $3.46\times10^{-3}$ |
| **HighFe.2** | $5.63\times10^{-3}$ | $2.55\times10^{-2}$ | $2.70\times10^{-2}$ | $2.78\times10^{-2}$ | $6.46\times10^{-3}$ | $7.38\times10^{-3}$ |
| **LowFe.2** | $2.07\times10^{-2}$ | $5.26\times10^{-2}$ | $4.59\times10^{-3}$ | $4.54\times10^{-3}$ | $2.85\times10^{-3}$ | $3.43\times10^{-3}$ |
| **HighFe.3** | $1.38\times10^{-2}$ | $4.38\times10^{-2}$ | $1.88\times10^{-2}$ | $2.25\times10^{-2}$ | $4.33\times10^{-3}$ | $5.66\times10^{-3}$ |
| **LowFe.3** | $3.71\times10^{-2}$ | $1.12\times10^{-1}$ | $4.73\times10^{-3}$ | $5.23\times10^{-3}$ | $3.82\times10^{-3}$ | $4.27\times10^{-3}$ |
| **HighFe.4** | $5.20\times10^{-3}$ | $2.20\times10^{-2}$ | $1.17\times10^{-2}$ | $1.16\times10^{-2}$ | $3.52\times10^{-3}$ | $4.88\times10^{-3}$ |
| **LowFe.4** | $2.81\times10^{-2}$ | $8.55\times10^{-2}$ | $4.63\times10^{-3}$ | $4.83\times10^{-3}$ | $1.40\times10^{-2}$ | $1.61\times10^{-2}$ |
| **HighFe.5** | $2.22\times10^{-2}$ | $8.15\times10^{-2}$ | $1.69\times10^{-2}$ | $1.66\times10^{-2}$ | $7.28\times10^{-3}$ | $9.27\times10^{-3}$ |
| **LowFe.5** | $2.84\times10^{-2}$ | $9.14\times10^{-2}$ | $4.89\times10^{-3}$ | $5.11\times10^{-3}$ | $2.60\times10^{-3}$ | $3.25\times10^{-3}$ |
| **p-value** | $4.42\times10^{-4}$* | | 0.122 | | 0.026* | |

| | ***Rhizomicrobium*** | | ***Desulfotomaculum*** | | ***Desulfobulbus*** | |
|---|---|---|---|---|---|---|
| **Sample** | Soil | Electrodes | Soil | Electrodes | Soil | Electrodes |
| **HighFe.1** | $5.81\times10^{-3}$ | $1.45\times10^{-3}$ | $2.18\times10^{-3}$ | $4.02\times10^{-3}$ | $7.74\times10^{-4}$ | $2.68\times10^{-3}$ |
| **LowFe.1** | $6.26\times10^{-3}$ | $1.64\times10^{-3}$ | $1.73\times10^{-3}$ | $3.44\times10^{-3}$ | $1.04\times10^{-3}$ | $2.68\times10^{-3}$ |
| **HighFe.2** | $1.03\times10^{-2}$ | $4.83\times10^{-3}$ | $2.69\times10^{-3}$ | $4.53\times10^{-3}$ | $2.42\times10^{-3}$ | $5.04\times10^{-3}$ |
| **LowFe.2** | $1.50\times10^{-2}$ | $7.34\times10^{-3}$ | $2.20\times10^{-3}$ | $3.82\times10^{-3}$ | $1.78\times10^{-3}$ | $3.31\times10^{-3}$ |
| **HighFe.3** | $1.06\times10^{-2}$ | $5.06\times10^{-3}$ | $2.19\times10^{-3}$ | $3.75\times10^{-3}$ | $7.34\times10^{-4}$ | $2.55\times10^{-3}$ |
| **LowFe.3** | $1.39\times10^{-2}$ | $7.48\times10^{-3}$ | $2.26\times10^{-3}$ | $4.77\times10^{-3}$ | $1.38\times10^{-3}$ | $3.44\times10^{-3}$ |
| **HighFe.4** | $1.10\times10^{-2}$ | $4.97\times10^{-3}$ | $1.80\times10^{-3}$ | $3.62\times10^{-3}$ | $9.63\times10^{-4}$ | $2.70\times10^{-3}$ |
| **LowFe.4** | $1.44\times10^{-2}$ | $5.62\times10^{-3}$ | $1.20\times10^{-2}$ | $1.61\times10^{-2}$ | $5.01\times10^{-3}$ | $7.86\times10^{-3}$ |
| **HighFe.5** | $9.71\times10^{-3}$ | $4.45\times10^{-3}$ | $2.66\times10^{-3}$ | $4.35\times10^{-3}$ | $9.70\times10^{-4}$ | $3.09\times10^{-3}$ |
| **LowFe.5** | $1.21\times10^{-2}$ | $4.91\times10^{-3}$ | $1.75\times10^{-2}$ | $2.41\times10^{-2}$ | $4.00\times10^{-3}$ | $6.80\times10^{-3}$ |
| **p-value** | $2.13\times10^{-7}$** | | 0.001* | | $2.36\times10^{-7}$* | |

(Table S3 continued)

| Sample | *Enterobacter* Soil | *Enterobacter* Electrodes | *Desulfovibrio* Soil | *Desulfovibrio* Electrodes | *Pseudomonas* Soil | *Pseudomonas* Electrodes |
|---|---|---|---|---|---|---|
| **HighFe.1** | $9.95\times10^{-4}$ | $7.38\times10^{-4}$ | $9.95\times10^{-4}$ | $1.66\times10^{-3}$ | $4.79\times10^{-4}$ | $9.08\times10^{-4}$ |
| **LowFe.1** | $4.44\times10^{-3}$ | $3.26\times10^{-3}$ | $1.54\times10^{-3}$ | $2.10\times10^{-3}$ | $4.07\times10^{-3}$ | $5.34\times10^{-3}$ |
| **HighFe.2** | $1.70\times10^{-2}$ | $1.01\times10^{-2}$ | $3.47\times10^{-3}$ | $3.99\times10^{-3}$ | $5.18\times10^{-4}$ | $1.18\times10^{-3}$ |
| **LowFe.2** | $3.14\times10^{-3}$ | $2.49\times10^{-3}$ | $6.19\times10^{-3}$ | $8.47\times10^{-3}$ | $3.25\times10^{-4}$ | $1.20\times10^{-3}$ |
| **HighFe.3** | $1.90\times10^{-3}$ | $1.02\times10^{-3}$ | $1.40\times10^{-3}$ | $2.38\times10^{-3}$ | $2.29\times10^{-3}$ | $3.19\times10^{-3}$ |
| **LowFe.3** | $1.77\times10^{-3}$ | $7.53\times10^{-4}$ | $1.79\times10^{-3}$ | $2.48\times10^{-3}$ | $8.95\times10^{-4}$ | $1.14\times10^{-3}$ |
| **HighFe.4** | $1.69\times10^{-3}$ | $8.72\times10^{-4}$ | $1.21\times10^{-3}$ | $2.57\times10^{-3}$ | $1.30\times10^{-3}$ | $2.28\times10^{-3}$ |
| **LowFe.4** | $1.50\times10^{-3}$ | $7.56\times10^{-4}$ | $2.93\times10^{-3}$ | $3.59\times10^{-3}$ | $1.46\times10^{-3}$ | $1.97\times10^{-3}$ |
| **HighFe.5** | $2.01\times10^{-3}$ | $1.50\times10^{-3}$ | $1.50\times10^{-3}$ | $2.02\times10^{-3}$ | $1.88\times10^{-3}$ | $2.55\times10^{-3}$ |
| **LowFe.5** | $1.13\times10^{-3}$ | $6.11\times10^{-4}$ | $2.24\times10^{-3}$ | $2.92\times10^{-3}$ | $2.94\times10^{-3}$ | $4.05\times10^{-3}$ |
| **p-value** | 0.057 | | 0.001* | | $3.42\times10^{-5}$* | |

| Sample | *Anaeromyxobacter* Soil | *Anaeromyxobacter* Electrodes | *Clostridium* Soil | *Clostridium* Electrodes | Sum of electrogenic bacteria Soil | Sum of electrogenic bacteria Electrodes |
|---|---|---|---|---|---|---|
| **HighFe.1** | $3.69\times10^{-4}$ | $2.15\times10^{-4}$ | $1.22\times10^{-3}$ | $2.55\times10^{-3}$ | $4.94\times10^{-2}$ | $7.78\times10^{-2}$ |
| **LowFe.1** | $2.30\times10^{-4}$ | $9.93\times10^{-5}$ | $3.18\times10^{-4}$ | $9.26\times10^{-4}$ | $2.99\times10^{-2}$ | $4.52\times10^{-2}$ |
| **HighFe.2** | $1.54\times10^{-3}$ | $1.59\times10^{-3}$ | $2.07\times10^{-4}$ | $6.87\times10^{-4}$ | $7.72\times10^{-2}$ | $9.27\times10^{-2}$ |
| **LowFe.2** | $9.88\times10^{-4}$ | $1.03\times10^{-3}$ | $7.28\times10^{-4}$ | $9.95\times10^{-4}$ | $5.84\times10^{-2}$ | $8.92\times10^{-2}$ |
| **HighFe.3** | $7.62\times10^{-4}$ | $5.26\times10^{-4}$ | $1.13\times10^{-4}$ | $5.26\times10^{-4}$ | $5.70\times10^{-2}$ | $9.09\times10^{-2}$ |
| **LowFe.3** | $9.27\times10^{-4}$ | $7.95\times10^{-4}$ | $4.71\times10^{-5}$ | $6.42\times10^{-4}$ | $6.86\times10^{-2}$ | $1.43\times10^{-1}$ |
| **HighFe.4** | $2.79\times10^{-4}$ | $2.18\times10^{-4}$ | $3.11\times10^{-5}$ | $5.54\times10^{-4}$ | $3.88\times10^{-2}$ | $5.63\times10^{-2}$ |
| **LowFe.4** | $5.85\times10^{-4}$ | $4.06\times10^{-4}$ | $5.44\times10^{-5}$ | $4.48\times10^{-4}$ | $8.46\times10^{-2}$ | $1.43\times10^{-1}$ |
| **HighFe.5** | $3.23\times10^{-4}$ | $3.30\times10^{-4}$ | $4.22\times10^{-5}$ | $5.85\times10^{-4}$ | $6.55\times10^{-2}$ | $1.26\times10^{-1}$ |
| **LowFe.5** | $2.60\times10^{-4}$ | $1.30\times10^{-4}$ | $2.05\times10^{-4}$ | $6.24\times10^{-4}$ | $7.62\times10^{-2}$ | $1.44\times10^{-1}$ |
| **p-value** | 0.014** | | $1.95\times10^{-4}$* | | $3.51\times10^{-4}$* | |

* two tail t-test, $p<0.05$, relative abundance among attached bacteria on the electrodes > relative abundance in soil.

** two tail t-test, $p<0.05$, relative abundance among attached bacteria on the electrodes < relative abundance in soil.

**Table S3.** Soil Fe (III) concentration at the different field sites.

| Site | Electrode set | Fe (III) (mM) |
|---|---|---|
| **Site 1-2** | Set 1<br>Set 2 | 1.797 |
| **Site 3-4** | Set 3<br>Set 4 | 0.351 |
| **Site 5-6** | Set 5<br>Set 6 | 0.761 |
| **Site 7-8** | Set 7<br>Set 8<br>Control | 2.282 |

**Table S4. A)** Details of attached bacteria on the electrodes and soil samples taken from the electrode pairs and control plates located at each field location and laboratory set up.

| Site | Electrode set and site sample | | Electrodes sample | | | Soil sample | | |
|---|---|---|---|---|---|---|---|---|
| | | | Sample name | Electrode surface area sampled | | Sample name | Soil wet mass | |
| | | | | Sample 1 ($cm^2$) | Sample 2 ($cm^2$) | | Sample 1 (g) | Sample 2 (g) |
| **Site 1-2** | Set 1 | Shallow electrode 1 | Elec. 1s | 9.375 | 9.375 | Soil 1s | 0.55 | 0.56 |
| | | 10 cm deep electrode 1 | Elec. 1.10 | 9.375 | 9.375 | Soil 1.10 | 0.56 | 0.56 |
| | Set 2 | Shallow electrode 2 | Elec. 2s | 9.375 | 9.375 | Soil 2s | 0.56 | 0.55 |
| | | 30 cm deep electrode 2 | Elec. 2.30 | 9.375 | 9.375 | Soil 2.30 | 0.55 | 0.55 |
| | Site 1-2 | Soil from site 1-2 | | | | Site 1-2 | 0.55 | 0.56 |
| **Site 3-4** | Set 3 | Shallow electrode 3 | Elec. 3s | 18.75 | 18.75 | Soil 3s | 0.55 | 0.55 |
| | | 10 cm deep electrode 3 | Elec. 3.10 | 9.375 | 9.375 | Soil 3.10 | 0.55 | 0.56 |
| | Set 4 | Shallow electrode 4 | Elec. 4s | 9.375 | 9.375 | Soil 4s | 0.55 | 0.55 |
| | | 30 cm deep electrode 4 | Elec. 4.30 | 9.375 | 9.375 | Soil 4.30 | 0.55 | 0.55 |
| | Site 3-4 | Soil from site 3-4 | | | | Site 3-4 | 0.55 | 0.56 |
| **Site 5-6** | Set 5 | Shallow electrode 5 | Elec. 5s | 9.375 | 9.375 | Soil 5s | 0.55 | 0.55 |
| | | 10 cm deep electrode 5 | Elec. 5.10 | 9.375 | 9.375 | Soil 5.10 | 0.55 | 0.56 |
| | Set 6 | Shallow electrode 6 | Elec. 6s | 9.375 | 9.375 | Soil 6s | 0.55 | 0.56 |
| | | 30 cm deep electrode 6 | Elec. 6.30 | 14 * | - | Soil 6.30 | 0.56 | 0.55 |
| | Site 5-6 | Soil from site 5-6 | | | | Site 5-6 | 0.56 | 0.55 |

* Deep electrode of set was only partially recovered, therefore only one sample was obtained from all faces.

(Table S4 A continued)

| Site | Electrode set and site sample | | Electrodes sample | | | Soil sample | | |
|---|---|---|---|---|---|---|---|---|
| | | | Sample name | Electrode surface area sampled | | Sample name | Soil wet mass | |
| | | | | Sample 1 ($cm^2$) | Sample 2 ($cm^2$) | | Sample 1 (g) | Sample 2 (g) |
| **Site 7-8 **** | Set 7 | Shallow electrode 7 | Elec. 7s | 37.5 | - | Soil 7s | 0.50 | - |
| | | 10 cm deep electrode 7 | Elec. 7.10 | 37.5 | - | Soil 7.10 | 0.50 | - |
| | Set 8 | Shallow electrode 8 | Elec. 8s | 37.5 | - | Soil 8s | 0.50 | - |
| | | 30 cm deep electrode 8 | Elec. 8.30 | 37.5 | - | Soil 8.30 | 0.50 | - |
| | Control | 10 cm deep plate | Plate. C.10 | 37.5 | - | Soil C.10 | 0.50 | - |
| | | 30 cm deep plate | Plate. C.30 | 37.5 | - | Soil C.30 | 0.50 | 0.50 |
| | Site 7-8 | Soil from site 7-8 | | | | Site 7-8 | 0.50 | 0.50 |
| **Lab** | Set 9 | Shallow electrode 9 | Elec. 9s | 18.75 | 18.75 | Soil 9s | 0.50 | 0.50 |
| | | 30 cm deep electrode 9 | Elec. 9.30 | 18.75 | 18.75 | Soil 9.30 | 0.50 | 0.50 |
| | Control | Shallow control | Plate. C.10 | 18.75 | 18.75 | Soil C.s | 0.50 | 0.50 |
| | | 30 cm deep plate | Plate. C.30 | 18.75 | 18.75 | Soil C.30 | 0.50 | 0.50 |

** Each set is the average of triplicate samples

**Table S4. B)** Details of attached bacteria on the electrodes and soil samples taken from constructed wetland mesocosms.

| **Depth (cm)** | **Electrode sample** | | **Soil sample near electrode** | |
|---|---|---|---|---|
| | **Sample name** | **Electrode surface area sampled ($cm^2$)** | **Sample name** | **Soil wet mass (g)** |
| **6** | Elec. High Fe.1 | 52.0 | Soil High Fe.1 | 0.352 |
| | Elec. Low Fe.1 | 52.0 | Soil Low Fe.1 | 0.334 |
| **12** | Elec. High Fe.2 | 52.0 | Soil High Fe.2 | 0.334 |
| | Elec. Low Fe.2 | 52.0 | Soil Low Fe.2 | 0.343 |
| **18** | Elec. High Fe.3 | 26.0 | Soil High Fe.3 | 0.372 |
| | Elec. Low Fe.3 | 26.0 | Soil Low Fe.3 | 0.384 |
| **24** | Elec. HighFe.4 | 26.0 | Soil High Fe.4 | 0.408 |
| | Elec. Low Fe.4 | 26.0 | Soil Low Fe.4 | 0.428 |
| **30** | Elec. High Fe.5 | 19.5 | Soil High Fe.5 | 0.458 |
| | Elec. Low Fe.5 | 26.0 | Soil Low Fe.5 | 0.409 |

**Table S5.** Diurnal cycle settings in the environmental growth chamber*

| Time | Temperature (°C) | Humidity (%) |
|---|---|---|
| 06:00 | 15.0 | 90 |
| 07:00 | 16.0 | 90 |
| 09:00 | 21.0 | 72 |
| 10:00 | 24.0 | 58 |
| 16:00 | 28.0 | 39 |
| 17:00 | 28.0 | 37 |
| 19:00 | 25.0 | 47 |
| 20:00 | 23.0 | 55 |

* Lights were on from 6:00 h to 20:00 h.

**a)** Recorded rainfall near Assunpink Wildlife Management Area (2016).

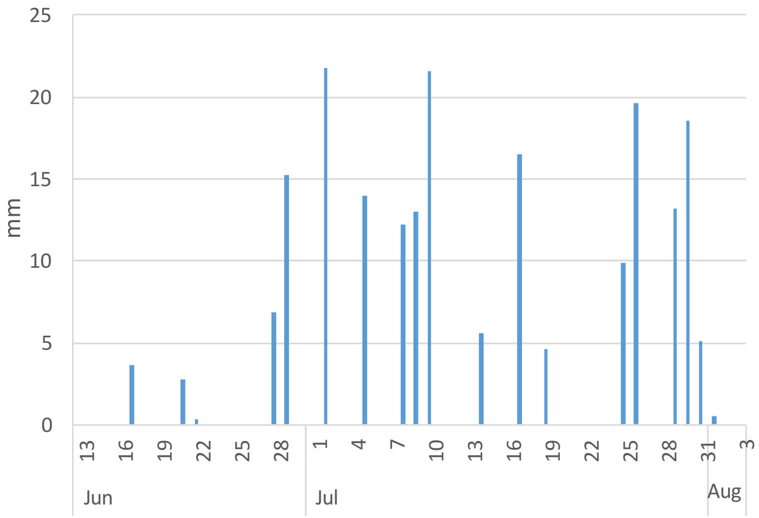


**b)** Recorded rainfall near Assunpink Wildlife Management Area (2018).

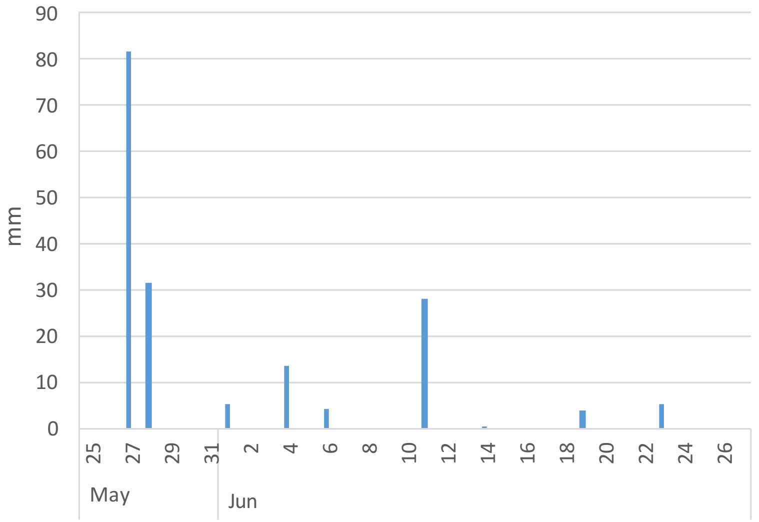


**Figure S1**. Daily rainfall record near Assunpink Wildlife Management Area during the time electrodes were deployed at the field site. **a)** Station US1NJMC0016 in Robbinsville Township 1.7 WSW, NJ. (GHCN-NOAA, 2018). **b)** Station: US USC00283951 in Hightstown 2 W, NJ (GHCN-NOAA, 2018).

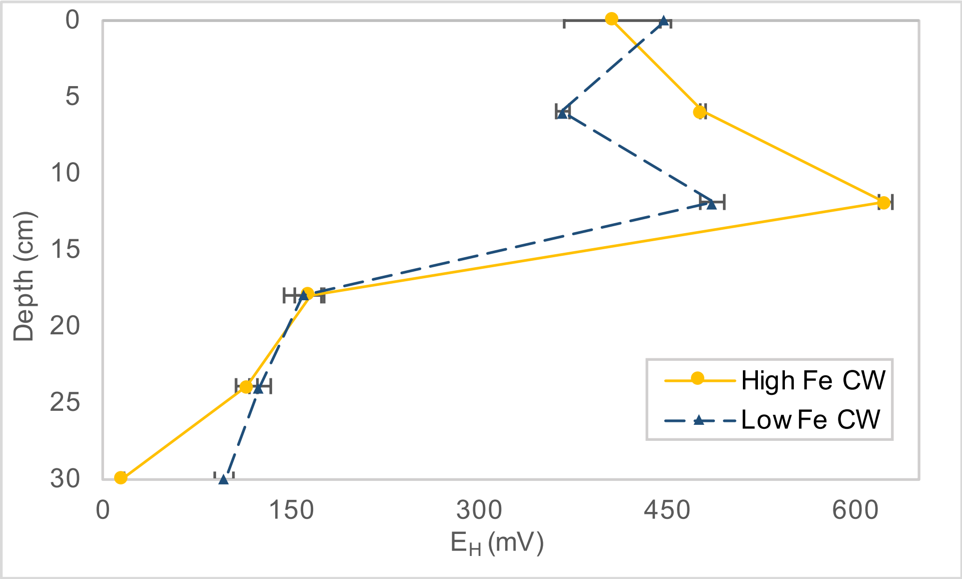


**Figure S2.** Oxidation-reduction Potential (ORP) vertical profiles in CW mesocosms on day 91. ORPs are expressed as $E_H$ (mV). Error bars are the standard deviation of measurements (n=3).

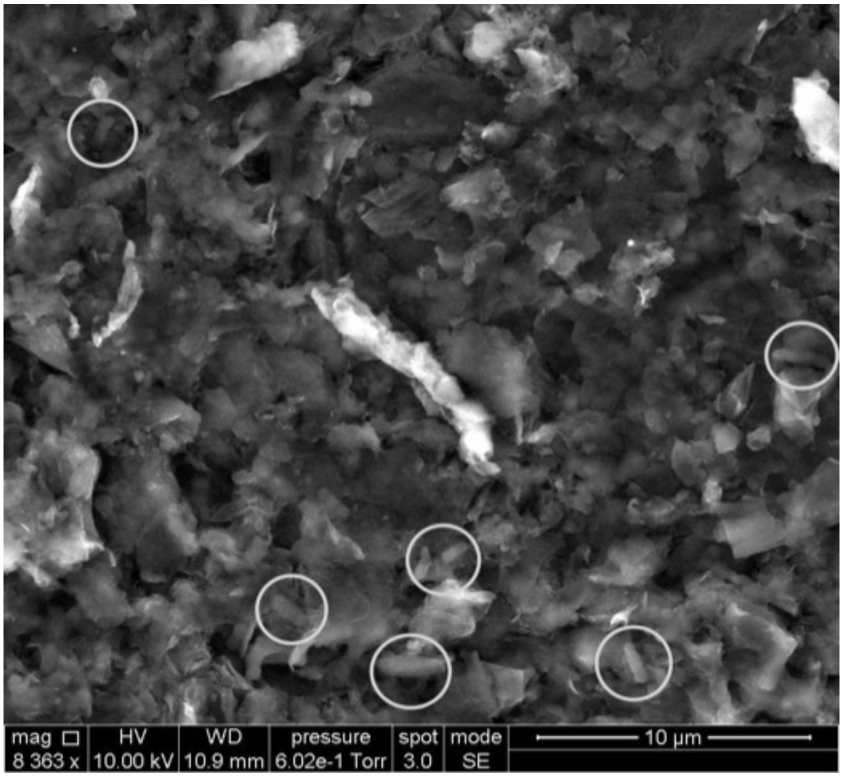


**Figure S3.** Scanning electron microscopy (SEM) image of the graphite electrode from a MEC inoculated with a pure culture of A6. The A6 cells that attached on the graphite plate are circled.

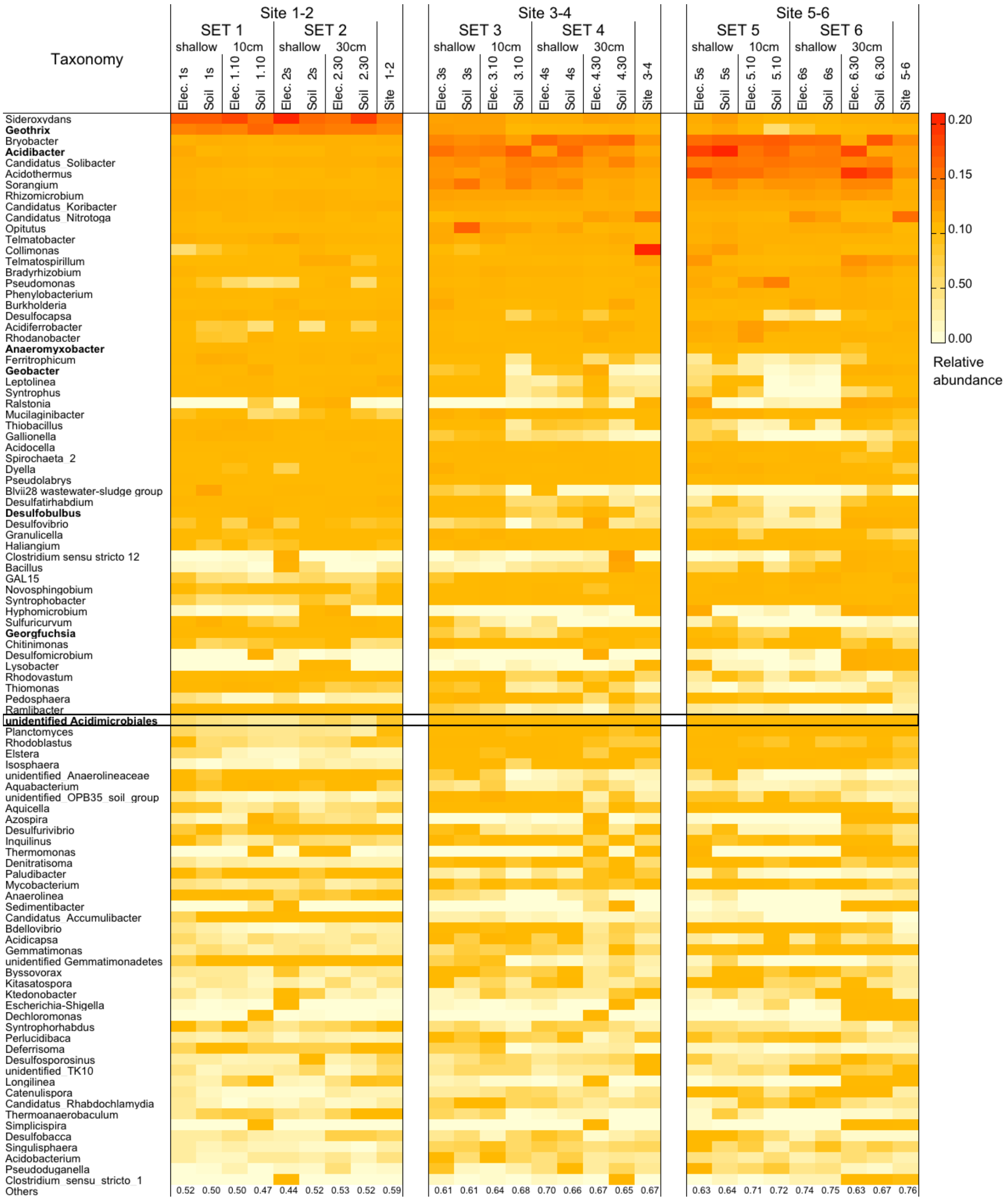


**Figure S4.** Relative abundance of the 100 most abundant genera from attached bacteria on the electrodes and on soil samples from the first group of electrode sets placed in the field. Acidimicrobiaceae sp. A6 had ≥ 97% identity with the unidentified Acidomicrobiales which ranked 56$^{th}$ in abundance. In bold other FeRB.

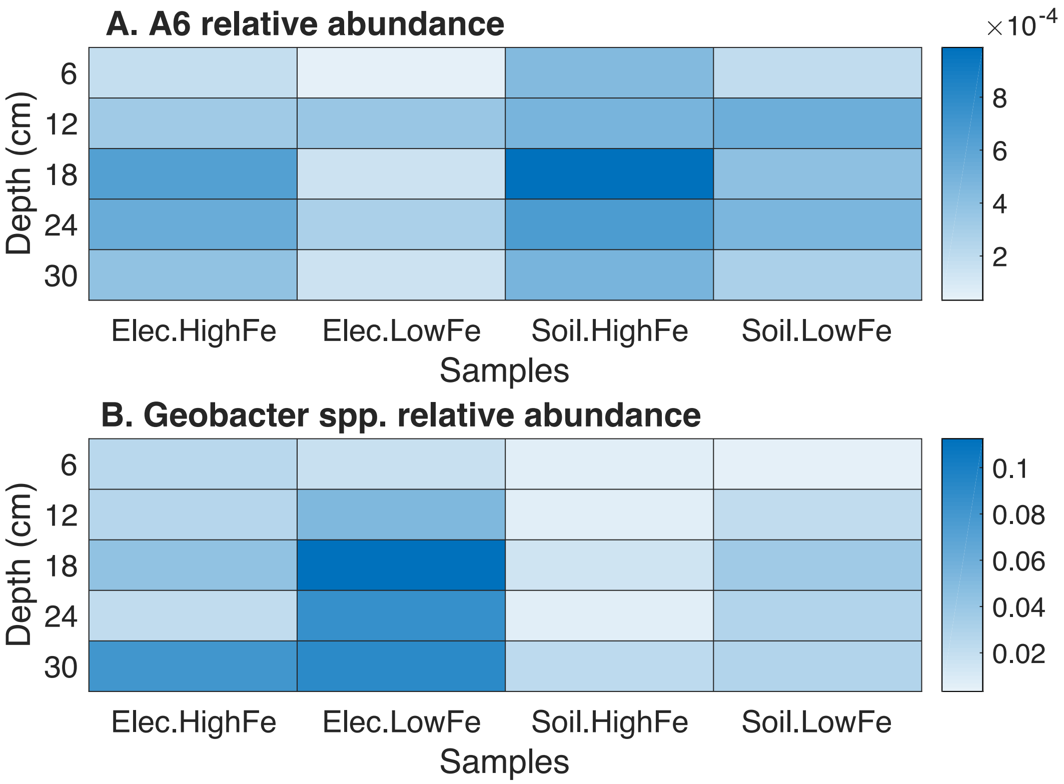


**Figure S5.** Relative abundance heat map of *Acidimicrobiaceae* sp. strain A6 (A) and *Geobacter* spp. (B) in CW mesocosm attached bacteria on the electrodes and soil samples.

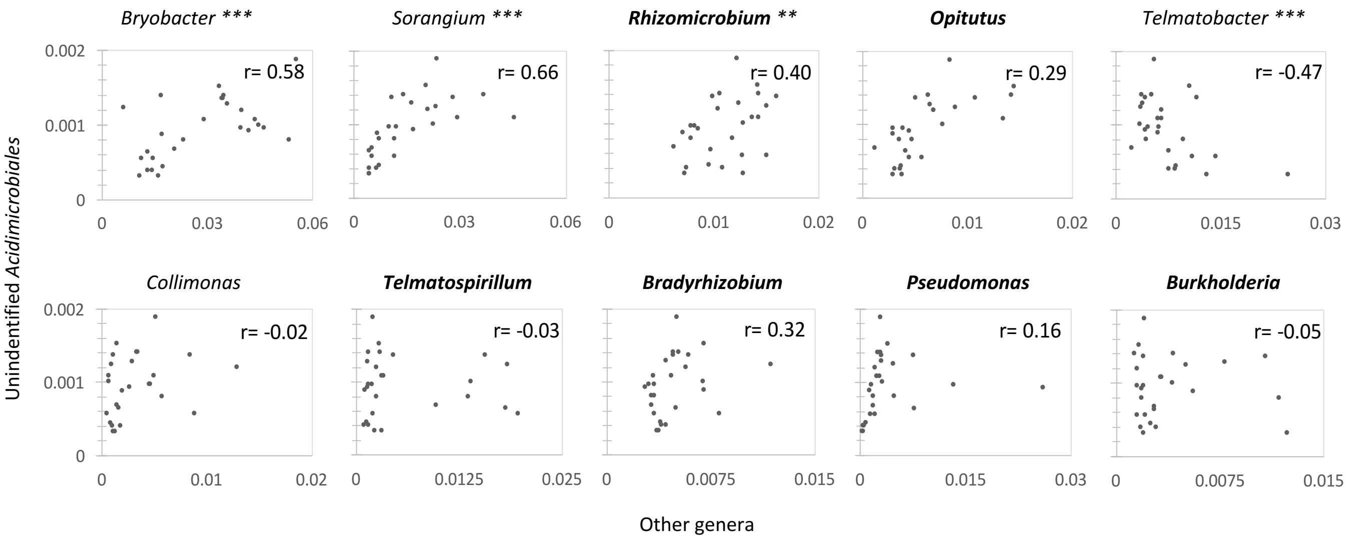


**Figure S6.** Correlation of the relative abundance between *Acidomicrobiaceae* sp. strain A6 (unidentified *Adicimicrobiales*) and other non-metal-reducing bacteria in attached bacteria on the electrodes and soil samples on the field-deployed electrodes (n=27). In bold nitrogen cycling bacteria. ***, P < 0.01; **, P < 0.5.
